# Proteomic Identification of Heat Shock-Induced Danger Signals in a Melanoma Cell Lysate Used in Dendritic Cell-Based Cancer Immunotherapy

**DOI:** 10.1155/2018/3982942

**Published:** 2018-03-18

**Authors:** Fermín E. González, Alexey Chernobrovkin, Cristián Pereda, Tamara García-Salum, Andrés Tittarelli, Mercedes N. López, Flavio Salazar-Onfray, Roman A. Zubarev

**Affiliations:** ^1^Laboratory of Experimental Immunology & Cancer, Faculty of Dentistry, Universidad de Chile, 8380492 Santiago, Chile; ^2^Millennium Institute on Immunology and Immunotherapy, Institute of Biomedical Sciences, Faculty of Medicine, Universidad de Chile, 8380453 Santiago, Chile; ^3^Department of Medical Biochemistry and Biophysics, Karolinska Institute, 17177 Stockholm, Sweden; ^4^Disciplinary Program of Immunology, Institute of Biomedical Sciences, Faculty of Medicine, Universidad de Chile, 8380453 Santiago, Chile; ^5^Laboratory of Molecular Virology, Departamento de Enfermedades Infecciosas e Inmunología Pediátrica, School of Medicine, Pontificia Universidad Católica de Chile, 8331150 Santiago, Chile; ^6^Millennium Institute on Immunology and Immunotherapy, Pontificia Universidad Católica de Chile, 8331150 Santiago, Chile

## Abstract

Autologous dendritic cells (DCs) loaded with cancer cell-derived lysates have become a promising tool in cancer immunotherapy. During the last decade, we demonstrated that vaccination of advanced melanoma patients with autologous tumor antigen presenting cells (TAPCells) loaded with an allogeneic heat shock- (HS-) conditioned melanoma cell-derived lysate (called TRIMEL) is able to induce an antitumor immune response associated with a prolonged patient survival. TRIMEL provides not only a broad spectrum of potential melanoma-associated antigens but also danger signals that are crucial in the induction of a committed mature DC phenotype. However, potential changes induced by heat conditioning on the proteome of TRIMEL are still unknown. The identification of newly or differentially expressed proteins under defined stress conditions is relevant for understanding the lysate immunogenicity. Here, we characterized the proteomic profile of TRIMEL in response to HS treatment. A quantitative label-free proteome analysis of over 2800 proteins was performed, with 91 proteins that were found to be regulated by HS treatment: 18 proteins were overexpressed and 73 underexpressed. Additionally, 32 proteins were only identified in the HS-treated TRIMEL and 26 in non HS-conditioned samples. One protein from the overexpressed group and two proteins from the HS-exclusive group were previously described as potential damage-associated molecular patterns (DAMPs). Some of the HS-induced proteins, such as haptoglobin, could be also considered as DAMPs and candidates for further immunological analysis in the establishment of new putative danger signals with immunostimulatory functions.

## 1. Introduction

Dendritic cells (DCs) are professional antigen presenting cells (APCs) that, upon encountering antigens (Ags) and proper sensing of danger signals, such as pathogen-associated molecular patterns (PAMPs) and/or damage-associated molecular patterns (DAMPs) in the tissue microenvironment, efficiently trigger adaptive immunity against pathogens and tumors [1–6], thus establishing a link between the innate and adaptive immunity [7]. Over the past decade, autologous DC-based immunotherapy against cancer has become a safe and reliable therapeutic approach, especially for solid tumors [8]. We have previously shown that immunotherapy using autologous *ex vivo*-generated tumor antigen presenting cells (TAPCells) from cytokine-activated monocytes (AM), and loaded with an original melanoma cell-derived lysate (referred to as TRIMEL), generated from three human melanoma cell lines, induces T cell-mediated immune responses and increased survival time of stage IV malignant melanoma (MM) patients [9–13]. In addition, more than 60% of treated patients showed a delayed type IV hypersensitivity (DTH) reaction against TRIMEL, indicating the development of an immunological memory. Importantly, positive DTH response correlated with prolonged survival of treated malignant melanoma patients [10–12]. Furthermore, we have observed that TAPCells vaccination induces differential response patterns of specific regulatory cell subpopulations in patients' peripheral blood leucocytes [10, 13]. These data strongly support an important role of TRIMEL in the *ex vivo* education of immunotherapeutic TAPCells and, in turn, in their capacity to trigger an *in vivo* antitumor immune response.

Despite these positive outcomes, around 40% of treated patients do not respond to the therapy (considering their DTH response) and have the same survival rate as nontreated ones [9–11]. This lack of response could be explained, at least in part, by carrying the 896 A>G *TLR4* gene polymorphism [12], an absence of sufficient immunogenic danger signals or a deficient timing in the input of danger signals to DCs [11], either during the *ex vivo* TAPCells generation or after their injection, which could induce deficiencies in migration, antigen processing, and/or presentation by inoculated cells.

*In vitro*, human DCs loaded with melanoma cells that were heat-treated at 42°C before being killed showed more efficient cross-priming to naive human CD8^+^ T cells than DCs loaded with unheated killed melanoma cells [14]. These heat-treated melanoma cells expressed enhanced amounts of the heat shock protein (HSP) 70, and the enhanced cross-priming could be reproduced by overexpression of Hsp70 in melanoma cells [14]. In this regard, we have previously shown that the TRIMEL lysate can induce a mature and committed DC phenotype from AM cells [11, 15]. Moreover, we have also demonstrated that the HS treatment of melanoma cells before their final lysis for TRIMEL generation increases calreticulin (CALR) plasma membrane translocation and induces the release of high mobility group box 1 (HMGB1) protein [11] and two well-described DAMPs [16, 17]. Importantly, *in vitro*-generated DCs from melanoma patients stimulated with TRIMEL induced a fivefold increase of IFN-*γ* release by a melanoma-specific cytotoxic T cell clone, compared to APCs stimulated with a non-HS-treated melanoma cell lysate [11], indicating the importance of the HS treatment in the capacity of TRIMEL to induce DCs with immunostimulatory properties. Both CALR and HMGB1 mobilizations were associated with enhanced DCs' maturation and with an efficient antigen cross-presentation capacity, respectively [11]. Additionally, HMGB1 from TRIMEL colocalizes with the receptor TLR4 on THP-1 cell surface, and the blockade of TLR4 in AM inhibits the expression of maturation-associated markers, proinflammatory cytokines, and CCR7 chemokine receptor induced by TRIMEL [12]. Moreover, DCs' ability to migrate to draining lymph nodes, a relevant prerequisite for its clinical efficacy, is also increased upon TRIMEL stimulation [18]. Taken together, these data strongly support that TRIMEL would contain not only HMGB1 and CALR but also other proteins or factors with DAMP functions, which contribute to its capacity to induce the TAPCells phenotype and their therapeutic performance. In this context, identifying the proteome changes in the lysate TRIMEL in response to HS would help to better understand TRIMEL's capacity to induce the *in vitro*/*ex vivo* DC maturation.

## 2. Material and Methods

### 2.1. Patients and Healthy Donors

Peripheral blood mononuclear cells (PBMC) were obtained by a leukapheresis procedure from four advanced (stage IV) MM patients previously treated using a reported TAPCells vaccination protocol [19]. Additionally, PBMC from six healthy donors, from the Blood Bank Service, Clinical Hospital, Universidad de Chile, were obtained. The present study was performed in agreement with the Helsinki Declaration and approved by the Bioethical Committee for Human Research of the Clinical Hospital, Universidad de Chile. All patients and healthy donors signed an informed consent form.

### 2.2. Cell Lines, Melanoma Cell Lysate TRIMEL, and HS Conditioning

The allogeneic cell lysate TRIMEL was prepared as previously described [10, 11]. Briefly, three different melanoma cell lines (MEL-1, MEL-2, and MEL-3), established from three tumor-infiltrated lymph nodes from metastatic HLA-A2^+^ stage IV melanoma patients and those positive for several melanoma-associated antigens, were cultured in RPMI-1640 medium (Gibco, Austria) supplemented with 10% (*v*/*v*) fetal bovine serum (FBS, Gibco/BRL), 10 *μ*g/mL streptomycin, and 100 mg/mL penicillin (Sigma, CA, USA), until 95% confluence. Cells were subcultured every 2-3 days. Before use, all the cell lines were tested by PCR techniques, to check the absence of potentially infecting virus or mycoplasma. The presence of contaminating bacteria was also ruled out by periodical culture testing in agar.

The cells were mixed in equal proportions (1 × 10^7^ cells for each cell line), resuspended in the therapeutic AIM-V medium (Gibco, CA, USA) at a concentration of 4 × 10^6^ cells/mL, HS-treated by incubating the cells one hour at 42°C, then two hours at 37°C, and finally lysed by performing three freeze-thaw cycles using liquid nitrogen. In order to perform the proteomic analysis, before the lysing step, part of the cell mixture was washed three times with PBS and frozen as pellets at −80°C until further proteomic analysis. Five independently produced batches for the complete lysate TRIMEL, with and without HS conditioning, were prepared (a total of 10 samples).

### 2.3. *In Vitro* Human DC Generation

PBMC of melanoma patients and healthy donors were cultured in serum-free therapeutic AIM-V medium at a concentration of 13 × 10^6^ cells/mL in six-well plates (BD Biosciences, Hershey, PA, USA) at 37°C and 5% CO_2_ for 2 hours. Thereafter, nonadherent cells were removed and the adherents (monocytes) were maintained and incubated for 22 additional hours in the presence of 500 U/mL recombinant human IL-4 (rhIL-4) and 800 U/mL of GM-CSF (US Biological, Swampscott, MA, USA). The obtained cytokine-activated monocytes (AM), which showed an immature DC-like phenotype, were then stimulated for 24 additional hours with 100 *μ*g/mL of TRIMEL or the lysate without HS conditioning.

### 2.4. Flow Cytometry Analysis

The cells were phenotypically characterized by flow cytometry using the following conjugated antibodies (Abs): mouse anti-human-HLA-ABC-FITC, HLA-DR-FITC, CD80-FITC, and CD11c-PE-Cy7 (eBioscience, San Diego, CA, USA). Briefly, cells were gently removed from the culture plates using cell scrapers. Then, the cells were centrifuged at 1000 rpm for 5 minutes at 4°C, washed with PBS, and incubated with Abs for 30 minutes. After being washed twice with PBS, samples were acquired on a FACSCalibur (BD Biosciences, Hershey, PA, USA) and analyzed using FlowJo software (Tree Star Inc., OR, USA). All the analyses were made in the CD11c^+^ cell population of each condition and sample.

### 2.5. Cell Lysis and Protein Extraction and Digestion

#### 2.5.1. Cell Lysis and Protein Extraction

A cell pellet containing 4 × 10^6^ cells was resuspended in 1 mL of lysis solution (0.2% ProteaseMax/10% acetonitrile (ACN)/50 mM ammonium bicarbonate (AmBic)). Cell lysis was performed over 10 minutes with the aid of rigorous vortexing. The lysate was kept at 95°C for 5 minutes and then subjected to 15 minutes sonication (30% amplitude, 3 : 3 pulse) with a Branson sonicator. Samples were centrifuged at 14,000 rpm over 7 minutes at room temperature and the precipitate was discarded. The total concentration of proteins was determined using a bicinchoninic acid assay (Pierce BCA assay kit, Thermo Fisher Scientific Inc.).

#### 2.5.2. In-Solution Digestion

Proteins were reduced by adding DTT to a final concentration of 10 mM and incubation for 30 minutes at 50°C, then alkylated via incubation with iodoacetamide for 30 minutes at room temperature. Proteins (80 *μ*g) were digested by adding 2 *μ*g of trypsin (Sequencing Grade Modified Trypsin, Promega) and incubated at 37°C for 9 hours. The digest was rigorously vortexed over 5 minutes. Digestion was terminated by the addition of 5% acetic acid. Samples were cleaned and desalted using C18 StageTips (Thermo Fisher Scientific Inc.), dried using a SpeedVac and resuspended in water with 0.1% formic acid.

### 2.6. Mass Spectrometry (MS)

Peptide mixture was injected into an Ultimate 3000 nanoflow LC system (Thermo Scientific, USA) in-line coupled to a Q Exactive mass spectrometer (Thermo Scientific). The chromatographic separation of the peptides was achieved using a 25 cm long in-house packed column (C18-AQ ReproSil-Pur®, Dr. Maisch GmbH, Germany) at 55°C with the following gradient: 4–30% ACN in 89 minutes, 26–95% ACN for 5 minutes, and 95% ACN for 8 minutes all at a flow rate of 250 nL/minutes.

The MS acquisition method comprised one full scan survey ranging from m/z 300 to m/z 1650 acquired with a resolution of *R* = 140,000 at m/z 200 and AGC target value of 5 × 10^6^, followed by data-dependent higher-energy collisional dissociation fragmentation scans from a maximum of 16 most intense precursor ions with a charge state ≥ 2. For dependent scans, the following parameters were used: precursor isolation width 4 Da, AGC target value of 2 × 10^5^, and normalized collision energy of 26. Scans were acquired in profile mode with a resolution of *R* = 17,500.

### 2.7. Protein Identification and Quantification

The MS raw data were analyzed with the MaxQuant software (version 1.5.3.30). A false discovery rate (FDR) of 0.01 for proteins and peptides and a minimum peptide length of six amino acids were required. Mass accuracy of the precursor ions was improved by the time-dependent recalibration algorithm of MaxQuant. The Andromeda search engine was used to search the MS/MS spectra against the Uniprot human database (containing 90,482 entries) combined with 262 common contaminants and concatenated with the reversed versions of all sequences. Enzyme specificity was set to trypsin. Further modifications were cysteine carbamidomethylation (fixed) as well as protein N-terminal acetylation, asparagine and glutamine deamidation, and methionine oxidation (variable). A maximum of two missed cleavages were allowed. Peptide identification was based on a search with an initial mass deviation of the precursor ion of up to 7 ppm. The fragment mass tolerance was set to 20 ppm on the m/z scale. Only proteins quantified with at least two peptides were considered for quantitation.

### 2.8. Bioinformatics and Statistical Analysis

Analysis of variance (ANOVA) and the Kruskal-Wallis test for nonparametric variables were used to compare significance of the differences in maturation marker expressions between studied groups. Differences were considered statistically significant at *p* < 0.05. The analyses were performed using GraphPad Prism 5 software (GraphPad Software Inc., USA).

Analysis of the data provided by MaxQuant was performed in the R scripting and statistical environment. Differences in relative protein abundances between heat-treated and control samples were assessed by moderated *t*-test using limma package [20]. Benjamini-Hochberg correction for multiple comparisons was used.

Gene set enrichment analysis and visualization of protein-protein interaction networks was performed using STRING software (http://string-db.org/) [21]. Each group of proteins—overexpressed, underexpressed, exclusively expressed in TRIMEL, and exclusively expressed in nontreated (no-HS) samples—was analyzed separately.

## 3. Results

### 3.1. The HS-Conditioning Contributes to the *In Vitro* Capacity of TRIMEL to Induce a Mature Phenotype on Human DCs

We have previously demonstrated that the addition of TRIMEL to primary human AM cells mediated up to fourfold induction of several surface markers associated with DC maturation such as MHC-I, MHC-II, CD80, CD83, and CD86 [11]. In addition, TRIMEL could also significantly induce a twofold increase in the expression of MHC-II, CD83, and CCR7 molecules in monocyte/macrophage THP-1 cells, generating a DC-like phenotype as compared with the unstimulated control cells [18].

**Figure 1:**
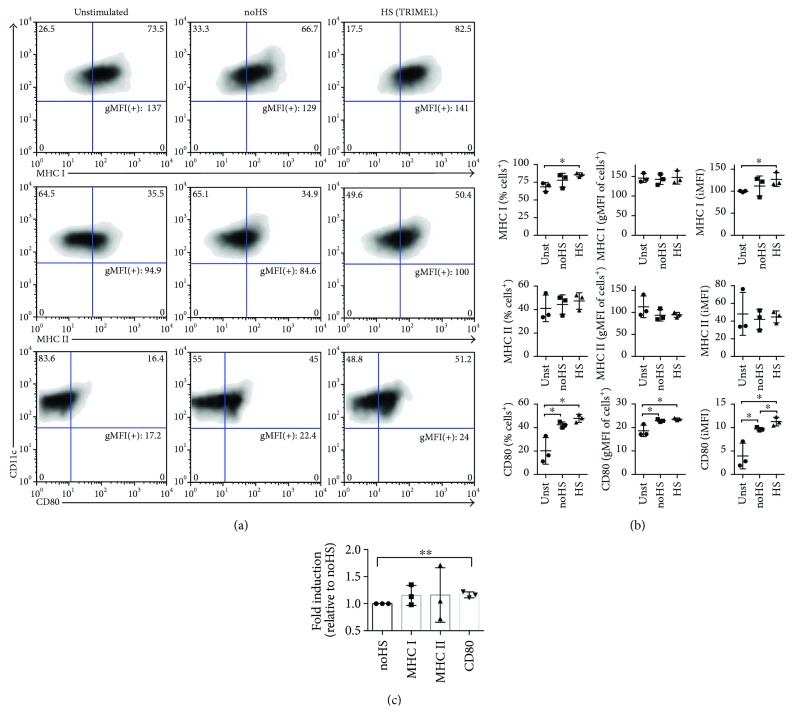
The HS conditioning of TRIMEL melanoma cells contributes to its *in vitro* DC maturation capacity. Representative density plots (a) and statistical quantification (b) of the DC-associated marker expression MHCI, MHCII, and CD80 in primary human cytokine-activated monocytes stimulated with TRIMEL (HS), or with the same lysate generated without heat shock conditioning (no-HS) (100 *μ*g/mL) or without lysate (unstimulated (Unst)). (b) The quantification of the maturation marker expression considered the % positive cells, the geometric mean fluorescence intensity (gMFI) of the positive cells, and the integrated MFI (iMFI: % positive cells × gMFI of positive cells/100). The expression of surface markers was assessed by flow cytometry (CD11c + cells were gated). Data represent three independent experiments with PBMC derived from three different stage IV MM patients. (c) Bars indicate the average fold induction and standard deviation (SD) of the iMFI of DC markers relative to monocytes stimulated with no-HS lysate. ^∗^ *p* < 0.05 and ^∗∗^ *p* < 0.01.

In order to evaluate the contribution of the HS conditioning of melanoma cells that generate TRIMEL to its capability in inducing a mature DC phenotype, we stimulated primary human AM cells with TRIMEL and the same lysate without the HS conditioning during 24 hours. All the canonical DC maturation-associated markers evaluated—MHC-I, MHC-II, and CD80—showed a higher percentage of positive cells in TRIMEL-stimulated cells when compared with control cells stimulated with the lysate generated with nontreated (no-HS) melanoma cells (Figure 1(a)). In addition, CD80 expression was significantly higher in cells stimulated with TRIMEL when compared with primary AM cells stimulated with nontreated melanoma cell-derived lysates (Figures 1(b) and 1(c)).

### 3.2. Proteomic Analysis of TRIMEL Showed Proteins Differentially Regulated by HS, Some of Them with Previously Described DAMP Function

**Figure 2:**
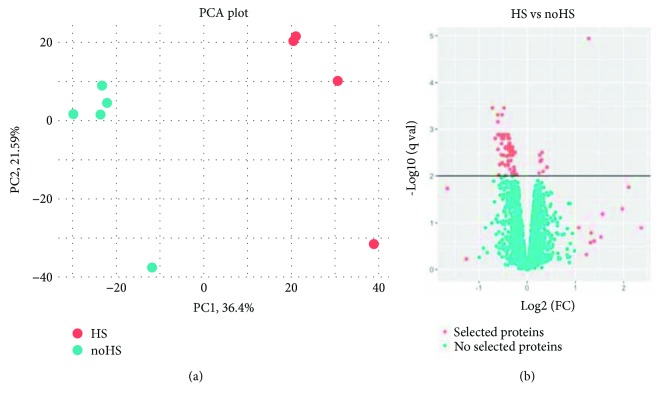
Quantitative proteomic comparison of TRIMEL lysates with (HS) and without (no-HS) heat shock conditioning. (a) Principal components analysis (PCA). (b) Volcano plot representation of moderated *t*-test analysis. Each point represents one protein plotted by log2 fold change (FC, average of four samples) versus minus logarithm of the *q*-value (Bemjamini-Hochberg corrected *p* value). The horizontal bar represents a *q*-value cutoff of 0.01. Red dots indicate the proteins selected for further analysis.

**Figure 3:**
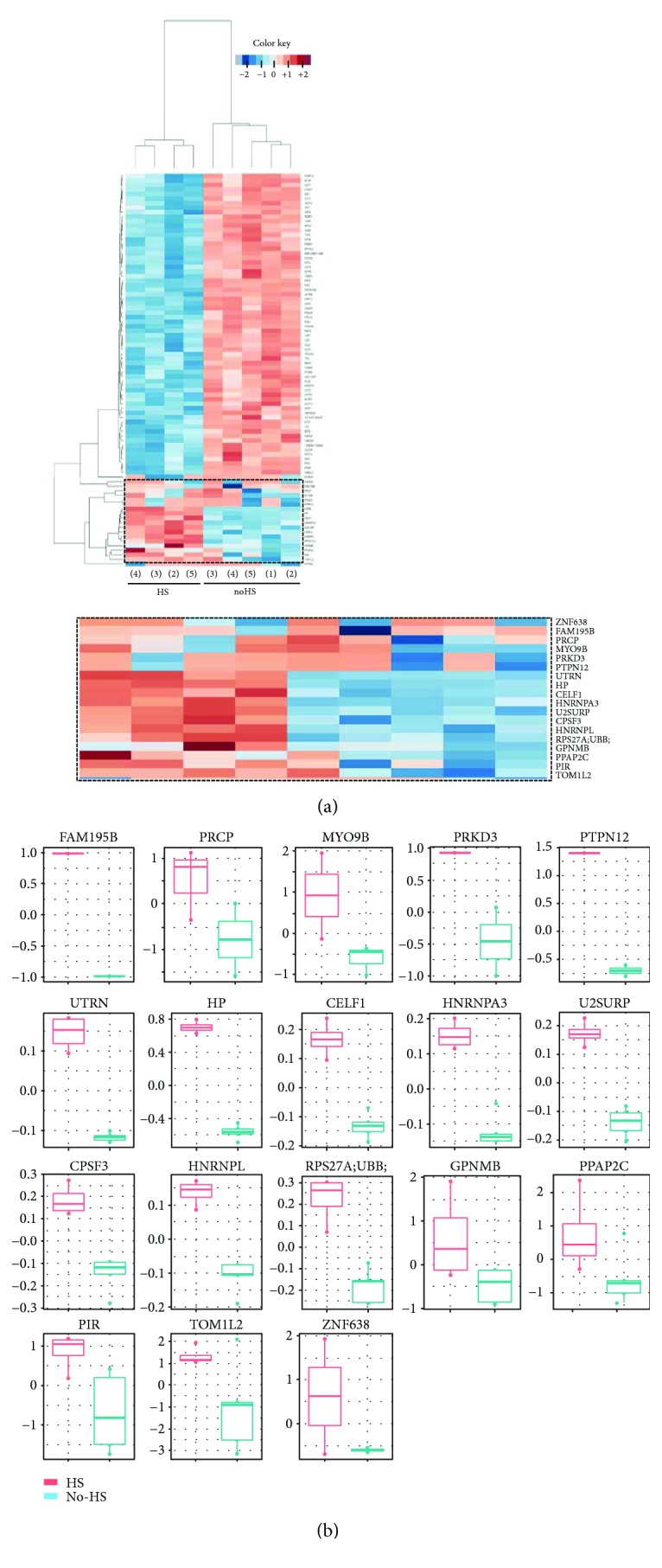
Expression profiles of selected proteins from TRIMEL and regulated by HS treatment. (a) Heat map with hierarchical clustering of proteins differentially expressed between TRIMEL (HS) and non-HS samples using a cutoff at *p* < 0.01. Protein names are displayed on the right and below is depicted an augmented section of the 18 HS-overexpressed proteins. Red, overexpressed; blue, underexpressed; and white, no change. The color-coded scale is indicated at the top of the chart. (b) Log-transformed relative protein expression of the 18 proteins regulated by HS treatment. The text and table only refer to 17 proteins because in one case a protein group was identified (RPS27A, UBB, UBC, UBA52, and UBBP4). It means the set of peptides matches all these five proteins and we cannot distinguish them here.

Proteomic analysis of the melanoma-derived lysate TRIMEL and nontreated (no-HS) lysates identified a total of 2798 proteins, 2740 of which were identified in both groups of samples, with and without HS conditioning (Supp. Figure 1). A principal component analysis clearly separates the samples by its HS conditioning (Figure 2(a)). In order to visualize changes in the protein expression induced by HS, proteomic data were visualized on a “volcano plot” (Figure 2(b)). Considering the regulated proteins by HS conditioning, a hierarchical clustering of proteins with the largest expression fold changes and *p* value < 0.01 was performed (Figure 3). As showed in Figure 3(a), a clearly distinctive protein expression profile for both groups of samples (TRIMEL (HS) and nontreated (no-HS) lysates) was found. Considering a selection criteria of *p* value < 0.01 or abs (log2 (FC)) > 1 as a cutoff, 18 proteins were selected as significantly more abundant in the melanoma-derived lysate TRIMEL (with HS conditioning) when compared with the nontreated (no-HS) samples (Figure 3(b) and Table 1), being haptoglobin (HP) one of the most overexpressed protein, since it fulfilled both selection criteria. Importantly, when analyzing this group, the protein U2 snRNP-associated SURP motif-containing protein (U2SURP) was found with previously described DAMP function [16, 17] (Table 2).

**Figure 4:**
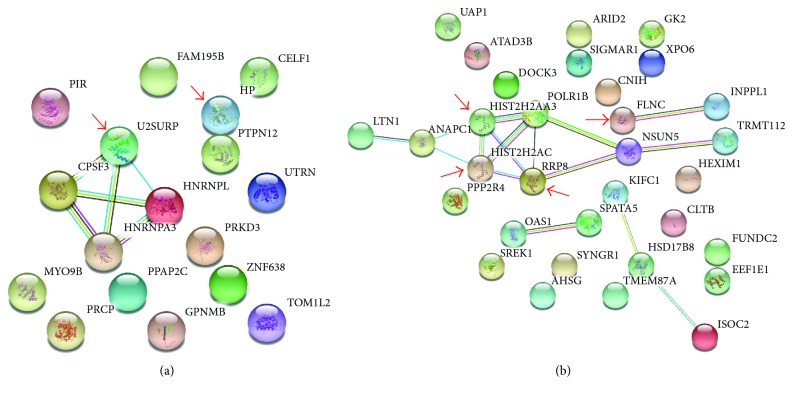
Overexpressed proteins by HS in the lysate TRIMEL. (a) Protein-protein interaction network of HS-overexpressed proteins from the lysate TRIMEL. Considering a cutoff *p* < 0.01 or abs (log2 (FC)) > 1, 18 proteins were significantly more abundant in TRIMEL lysate compared to no-HS-treated lysates; however, only 17 were considered for STRING analysis. Red arrows indicate the top one overexpressed protein HP and U2SURP a previously described DAMP protein. (b) Protein-protein interaction network of the 32 proteins exclusively identified in TRIMEL samples. Red arrows indicate the exclusively identified DAMPs (HIST2H2AC, HIST2H2AA3, RRP8, and FLNC) in TRIMEL samples. Line color indicates the type of interaction evidence. Known interactions: cyan, from curated databases and pink, experimentally determined. Predicted interactions: green, gene neighborhood. Others: yellow, textmining; black, coexpression. Interaction confidence score, 0.4 (medium).

STRING analysis of protein interactions among the overexpressed proteins, showed protein-protein relationships just among the proteins U2SURP, CPFS3, HNRNPL, and HNRNPA3 (Figure 4(a)). Of note, HP is not involved in the cluster of protein interaction identified by our analysis. This analysis was done considering only 17 proteins because in one case, a group of proteins was identified (proteins RPS27A, UBB, UBC, UBA52, and UBBP4). It means that the set of peptides matches all these proteins so we could not distinguish among them.

On the other hand, 73 proteins were significantly less abundant in TRIMEL (HS-conditioned) samples compared with non-HS-conditioned ones (Supp. Table 1). Of note, among this group of proteins, heat shock protein family A (Hsp70) member 4 (HSPA4) and ribosomal protein S19 (RPS19) are proteins previously described as DAMPs (Supp. Table 1). Remarkably, HMGB1, a well-known protein with an extensively described DAMP function, did not change in its abundance by HS-conditioning. Of note, protein-protein interaction analysis by STRING showed direct interaction between RPS19 and proteins from the translational machinery like ETF1, BTF3, EEF2, EIF1, EIF3J, and EIF4E proteins (Supp. Figure 2).

### 3.3. Expression Profile Analysis of TRIMEL Showed Proteins Exclusively Identified in TRIMEL and in No-HS-Conditioned Lysates

Our proteomic analysis also revealed that there was a group of 32 proteins only identified in the lysate TRIMEL (HS-conditioned) (Table 3). Among proteins only identified in TRIMEL samples, histone cluster 2 H2A family member c (HIST2H2AC) and histone cluster 2 H2A family member a3 (HIST2H2AA3) have been previously described to possess DAMP function (Table 2). STRING analysis showed the direct interaction between these two proteins with ANAPC1, RRP8, and POLR1B and indirectly with LTN1, NSUN5, and TRMT112 (Figure 4(b)). In addition, a group of 26 proteins were only identified in nontreated (no-HS) samples (Supp. Table 2). Notably, when we analyzed the group of proteins exclusively identified in nontreated samples (no-HS), we did not find proteins with reported DAMP function. STRING analysis of this group of proteins showed a main interaction group among proteins WDR82, PPP1R2, PPP3CB, PPP3CA, and EPS15 (Supp. Figure 3).

## 4. Discussion

During recent years, intact cancer cells and cancer cell-derived lysates have been extensively used in different cell-based immunotherapies against cancer. This is mainly because they constitute not only a broad source for tumor-associated antigens but also for several and biochemically diverse molecules with immunomodulatory activity. Indeed, *ex vivo* educated DCs using tumor cell-derived lysates have become an important approach in cancer immunotherapy, especially in the treatment of solid tumors [8]. We have previously demonstrated the capacity of the allogeneic HS-conditioned lysate TRIMEL to induce a mature DC phenotype on *ex vivo* generated TAPCells. In turn, it is able to trigger an *in vivo* antitumor immunity in advanced MM patients [10–12]. In this context, characterization of the proteomic profile changes induced by HS would help to identify more proteins and protein-protein interactions involved in DC maturation process triggered by their stimulation with cancer cell lysates.

Here, we showed that HS conditioning of melanoma cancer cells belonging to TRIMEL is responsible, at least in part, for the TRIMEL maturation capacity on DC phenotype. In this regard, in a previous study, we have shown that HS conditioning is able to induce the secretion of the DAMP protein HMGB1 by melanoma cells as well as the mobilization of CALR to plasma membrane, a well-known “eat me” signal for phagocytic cells [11]. In the current study, CALR was found among proteins slightly overexpressed after HS conditioning (*p* value = 0.0259; logFC = 0.25), suggesting that HS treatment not only mobilizes this protein towards the plasma membrane of melanoma cells but also induces its expression by these cells. However, the nuclear protein HMGB1 did not change its abundance upon HS (*p* value = 0.5610; logFC = −0.14), indicating that this stimulus is only able to induce its secretion but not its expression by melanoma cells.

Interestingly, melanoma cells upon HS treatment underexpressed more proteins than the ones they overexpressed. This observation could be explained, at least in part, by the fact that HS constitutes a stress factor and, therefore, cells under HS enter in a metabolic state that can alter cellular protein homeostasis. In this context, Hsp70 has been involved in the modulation of the protein synthetic machinery, switching from a degradation phase to the protein synthesis phase [22]. Here, we found proteins belonging to the HSP family differentially regulated by HS. Indeed, heat shock protein family A (Hsp70) member 4 (HSPA4) was significantly underexpressed upon HS treatment and, on the contrary, heat shock protein family A (Hsp70) member 9 (HSPA9), heat shock protein family D (Hsp60) member 1 (HSPD1), and heat shock protein family E (Hsp10) member 1 (HSPE1) were slightly overexpressed. Related with this, one of the significantly underexpressed proteins was PSME1 (proteasome activator complex subunit 1), which is a regulator of proteasome activity [23], suggesting that HS treatment inhibits protein degradation in melanoma cells and, in turn, can contribute to modify protein homeostasis. In addition, several proteins involved in translational machinery, like EIF1, EIF3J, and EIF4E, and different 40S ribosomal proteins are among the underexpressed group of proteins. A less abundance of these proteins could contribute to the inhibition of the translation of different downstream proteins. Interestingly, some of these translation factors, like EIF4E, have been described to be downregulated under heat stress response during exercise [24]. On the other hand, the group of proteins overexpressed/exclusive in no-HS samples could be also relevant to be analyzed. Indeed, transcription factor binding to IGHM enhancer 3 (TFE3) has been also associated with stress response by promoting cell adaptation to nutrient deprivation by upregulating transcription of numerous autophagic and lysosomal genes [25].

The main protein-protein interaction among overexpressed proteins involves U2SURP, CPFS3, HNRNPL, and HNRNPA3 proteins. HNRNPL (heterogeneous nuclear ribonucleoprotein L) and HNRNPA3 (heterogeneous nuclear ribonucleoprotein A3) are members of the HNRNP family that regulate different pre-mRNA and mature mRNA transcription [26]. Importantly, HNRNPL has been recently associated with aggressiveness and poor prognosis in different malignances such as colorectal cancer, hepatocellular carcinoma, and bladder cancer [27–29]. On the contrary, and without interactions with other overexpressed proteins, PTPN12 (tyrosine-protein phosphatase nonreceptor type 12) is a tumor suppressor protein and has been associated with overall survival in esophageal squamous cell carcinoma patients and non-small-cell lung cancer [30, 31].

Currently, and despite the high research activity in this field, there is no consensus about DAMPs' immunomodulatory effects (i.e., promoting either antitumor immunity or cancer progression), as well as whether they can be divided based on the timing of their functions on APCs: early-stage effect-related DAMPs, that is, DAMPs inducing chemotaxis, phagocytosis, and proinflammatory cytokine production; or late-stage effect-related DAMPs, that is, DAMPs inducing migration, costimulatory molecules expression, and tumor-associated antigen cross-presentation. In this context, TRIMEL could be considered a source for initial danger signals (or early-stage DAMPs) to be sensed by immature DC which, in turn, are able to sense further signals *in vivo* after its injection into MM patients. Additionally, and in line with the concept recently coined by Yatim and colleagues [32], the DAMPs carried by TRIMEL could be considered as both inducible DAMPs (iDAMPs) and constitutive DAMPs (cDAMPs). Indeed, the six proteins from TRIMEL with described/putative DAMP function (two from the overexpressed group of proteins and four from proteins exclusively identified in TRIMEL) as well as CALR can be considered as an example of iDAMPs, and HMGB1, previously described as being also relevant for TRIMEL properties [11], could be considered as a cDAMP. These proteins contribute, probably in a synergic way, to the ability of the lysate TRIMEL to *ex vivo* induce a mature phenotype in therapeutic DCs (TAPCells) and could be responsible, at least in part, for the clinical effect of these cells in treated MM patients.

One of the main overexpressed proteins by HS conditioning of the melanoma cells belonging the lysate TRIMEL was HP, a plasmatic glycoprotein with a molecular weight of 38 kDa. The main function of HP is binding haemoglobin (Hb), forming a stable complex HP-Hb, which is cleared via CD163-mediated endocytosis and thus preventing the oxidative tissue damage induced by free haemoglobin [33, 34]. In fact, this protein-protein interaction described between HP and Hb was also confirmed by our STRING analysis, where the only interaction of HP was with HBD and HBB proteins. Moreover, it has been described that HP has a protective role in T cell-mediated inflammatory skin diseases [35]. In addition, it has been previously suggested as a biomarker for early diagnosis in ovarian cancer [36, 37], and its fucosylated form is considered a diagnosis and postsurgical prognosis biomarker in pancreatic and colorectal cancer, respectively [38, 39]. Importantly, during the last years, the capacity of HP to activate DCs was shown in a murine skin transplantation model [40], and recently, the same group showed an amplifying role of HP in inflammation after cardiac transplantation in a murine model, demonstrating a relevant interaction between this protein and the immune system [41]. Interestingly, HP also binds to HMGB1 forming a HP-HMGB1 complex, which elicits the secretion of anti-inflammatory enzymes (e.g., heme oxygenase-1) and cytokines (e.g., IL-10) in WT but not in CD163-deficient macrophages [42], indicating a regulatory function of HP. In this context, in order to confirm HP as a DAMP molecule, further experiments should be focused on the interaction of HP with immune receptors, such as pattern recognition receptors (PRRs), with APCs and other immune cell types.

This study constitutes a conceptual approach in order to identify DAMPs that are induced by HS, which is a fundamental step in TRIMEL generation and in its capacity to induce *ex vivo*/*in vitro* DC maturation. We have shown that the clinically used lysate TRIMEL carries at least six proteins with previously described or putative DAMP function. These proteins, induced by HS conditioning of the melanoma cells before their lysis for TRIMEL generation, could be considered as iDAMPs and, therefore, involved in the capacity of TRIMEL to induce the *ex vivo* maturation of TAPCells and their *in vivo* clinical performance in vaccinated patients [10]. Importantly, there are several other proteins in the lysate that have been over- or exclusively expressed upon HS treatment and, therefore, are potential candidates to be confirmed as DAMPs such as HP. DC maturation is a very complex process, which strongly depends on the amount and quality of different signals that are sensed by DCs from either physiologic and pathologic microenvironments [2, 8]. Biochemically, some of these signals are proteins, nucleic acids, metabolites, and extracellular matrix-derived molecules, among others, constituting an even more complex scenario. Related to this, the lysate TRIMEL must contain several nonprotein factors that also contribute to its capacity to induce DC maturation. However, the specific contribution of these factors on TRIMEL capacity to induce the *ex vivo*/*in vitro* DC maturation is still unknown. Further analysis focused on the determination of the amount and relative contribution of different DAMPs in inducing a mature phenotype in human DC by clinically used cancer cell-derived lysates would help to design new strategies for efficiently activating *ex vivo*-generated DCs and, in turn, developing more effective DC-based immunotherapies against cancer.

## Figures and Tables

**Table 1:** Currently known functions of selected gene-proteins upregulated by HS. The protein lists was previously filtered by *p* < 0.01 or abs (log2(FC)) > 1.

Gene	ID (NCBI)	Full name (NCBI)	Function (gene ontology)	Reference
CELF1	10658	CUGBP Elav-like family member 1	(i) BRE; RNA; mRNA; pre-mRNA; protein and translation repressor activity, nucleic acid binding	[43–48]

CPSF3	51692	Cleavage and polyadenylation specific factor 3	(i) Protein binding	[49]

FAM195B	348262	MAPK regulated corepressor interacting protein 1	(i) Protein binding	[50, 51]

GPNMB	10457	Glycoprotein NMB	(i) Chemoattractant and receptor ligand activity	[52–54]
			(ii) Heparin; protein and syndecan binding	

HNRNPA3	220988	Heterogeneous nuclear ribonucleoprotein A3	(i) RNA and protein binding	[44, 45, 55]

HNRNPL	3191	Heterogeneous nuclear ribonucleoprotein L	(i) RNA; pre-mRNA; protein and transcription regulatory region DNA binding	[44, 45, 56–58]

HP	3240	Haptoglobin	(i) Hemoglobin and protein binding	[59, 60]

MYO9B	4650	Myosin IXB	(i) ATPase; GTPase activator; microfilament motor and NOT protein homodimerization activity	[61–65]
			(ii) ADP; ATP; Rho GTPase; Roundabout; actin; calmodulin and protein binding	

PIR	8544	Pirin	(i) Quercetin 2,3-dioxygenase and transcription cofactor activity	[66–69]
			(ii) Metal ion and protein binding	

PPAP2C	8612	Phospholipid phosphatase 2	(i) Phosphoprotein phosphatase activity	[70, 71]
			(ii) Protein binding	

PRCP	5547	Prolylcarboxypeptidase	(i) Protein binding	[72]

PRKD3	23683	Protein kinase D3	(i) Kinase activity	[73, 74]
			(ii) Protein binding	

PTPN12	5782	Protein tyrosine phosphatase, nonreceptor type 12	(i) Nonmembrane spanning protein tyrosine phosphatase; phosphoprotein phosphatase and protein tyrosine phosphatase activity	[75–80]
			(ii) SH3 domain and protein binding	

TOM1L2	146691	Target of myb1 like 2 membrane trafficking protein	(i) Clathrin; protein and protein kinase binding	[81, 82]

U2SURP	23350	U2 snRNP associated SURP motif-containing protein	(i) RNA and protein binding	[45, 83]

UTRN	7402	Utrophin	(i) Actin; integrin; protein; protein kinase and vinculin binding	[84, 85]

ZNF638	27332	Zinc finger protein 638	(i) RNA and double-stranded DNA binding	[45, 86]

ID, identification number; NCBI, National Center for Biotechnology Information.

**Table 2:** Protein with related/putative DAMP functions that were overexpressed and exclusively expressed in TRIMEL treated with HS.

Gene	ID (NCBI)	Full name (NCBI)	Protein subgroup	Reference
FLNC	2318	Filamin C	Exclusive	[87]
HIST2H2AA3/HIST2H2AC	8337/8338	Histone cluster 2 H2A family member a3/Histone cluster 2 H2A family member c	Exclusive	[17]
HP	3240	Haptoglobin	Overexpressed	[42]
RRP8	23378	Ribosomal RNA processing 8, methyltransferase, homolog (yeast)	Exclusive	[88]
U2SURP	23350	U2 snRNP associated SURP domain containing	Overexpressed	[17]

ID, identification number; NCBI, National Center for Biotechnology Information.

**Table 3:** Currently known functions of gene-proteins exclusively identified in HS-conditioned samples (TRIMEL).

Gene	ID (NCBI)	Full name (NCBI)	Function (gene ontology)	Reference
AHSG	197	Alpha 2-HS glycoprotein	(i) Kinase inhibitor activity	[89]
ANAPC1	64682	Anaphase promoting complex subunit 1		

ARID2	196528	AT-rich interaction domain 2	(i) Protein binding	[90]

ATAD3B	83858	ATPase family, AAA domain containing 3B		

CLTB	1212	Clathrin light chain B	(i) Protein binding	[91]

CNIH	10175	Cornichon family AMPA receptor auxiliary protein 1		

DOCK3	1795	Dedicator of cytokinesis 3	(i) Protein binding	[92]

EEF1E1	9521	Eukaryotic translation elongation factor 1 epsilon 1	(i) Protein binding	[93]

FLNC	2318	Filamin C	(i) Ankyrin; cytoskeletal protein and protein binding	[94–96]

FUNDC2	65991	FUN14 domain containing 2		

GK2	2712	Glycerol kinase 2	(i) Glycerol kinase activity	[97]

HEXIM1	10614	Hexamethylene bisacetamide inducible 1	(i) Cyclin-dependent protein serine/threonine kinase inhibitor activity	[98–101]
			(ii) 7SK snRNA; protein and snRNA binding	

HIST2H2AA3/HIST2H2AC	8337/8338	Histone cluster 2 H2A family member a3/Histone cluster 2 H2A family member c	

HSD17B8	7923	Hydroxysteroid 17-beta dehydrogenase 8	(i) 3-Hydroxyacyl-CoA dehydrogenase; 3-oxoacyl-[acyl-carrier-protein] reductase (NADH) and estradiol 17-beta-dehydrogenase activity	[102–104]
			(ii) NADH and protein binding	

INPPL1	3636	Inositol polyphosphate phosphatase like 1	(i) SH2 domain and protein binding	[105, 106]

ISOC2	79763	Isochorismatase domain containing 2	(i) Protein binding	[107]

KIFC1	3833	Kinesin family member C1	(i) Microtubule motor activity	[108]
			(ii) ATP binding	

LTN1	26046	Listerin E3 ubiquitin protein ligase 1	(i) Protein binding	[109]

NSUN5	55695	NOP2/Sun RNA methyltransferase family member 5	(i) RNA binding	[44, 45]

OAS1	4938	2′-5′−Oligoadenylate synthetase 1	(i) 2′-5′-Oligoadenylate synthetase activity	[93, 110–112]
			(ii) ATP; double-stranded RNA and protein binding	

POLR1B	84172	RNA polymerase I subunit B	(i) Protein binding	[113]

PPP2R4	5524	Protein phosphatase 2 phosphatase activator	(i) Contributes to ATPase; protein heterodimerization; protein homodimerization; protein phosphatase regulator and protein tyrosine phosphatase activator activity	[93, 114–116]
			(ii) ATP; protein, protein phosphatase 2A and receptor binding	

RRP8	23378	Ribosomal RNA processing 8, methyltransferase, homolog (yeast)	(i) S-Adenosylmethionine-dependentmethyltransferase activity	[44, 45, 117]
			(ii) RNA; methylated histone and protein binding	

SIGMAR1	10280	Sigma nonopioid intracellular receptor 1	(i) Drug binding	[118]

SPATA5	166378	Spermatogenesis associated 5		

SREK1	140890	Splicing regulatory glutamic acid and lysine rich protein 1	(i) RNA and protein binding	[45, 93]

SYNGR1	9145	Synaptogyrin 1	(i) Protein binding	[93]

TMEM87A	25963	Transmembrane protein 87A		

TRMT112	51504	tRNA methyltransferase 11-2 homolog (*S. cerevisiae*)	(i) Protein methyltransferase activity	[119–121]
			(ii) Protein binding	

UAP1	6675	UDP-N-acetylglucosamine pyrophosphorylase 1	(i) Identical protein binding	[93]

XPO6	23214	Exportin 6	(i) Protein transporter activity	[122]
			(ii) Protein binding	

ID, identification number; NCBI, National Center for Biotechnology Information.

## Supplementary Materials

Supplementary Materials Supplementary Figure 1: Venn-diagram illustrating the number of identified proteins. Supplementary Figure 2: protein-protein interaction network from downregulated proteins by HS in the lysate TRIMEL. Supplementary Figure 3: protein-protein interaction network of proteins exclusively expressed in no-HS-conditioned melanoma-derived lysates samples. Supplementary Table 1: currently known functions of selected gene-proteins downregulated by HS. Supplementary Table 2: currently known functions of gene-proteins exclusively expressed by non-HS samples. Supplementary list of references.
