# Supplementary material for: Proteomic Identification of Heat Shock-Induced Danger Signals in a Melanoma Cell Lysate Used in Dendritic Cell-Based Cancer Immunotherapy
Source: J Immunol Res. 2018 Mar 18;2018:3982942. doi: 10.1155/2018/3982942 (PMC5878886; doi:10.1155/2018/3982942)
Supplement: Supplementary Materials — Supplementary Figure 1: Venn-diagram illustrating the number of identified proteins. Supplementary Figure 2: protein-protein interaction network from downregulated proteins by HS in the lysate TRIMEL. Supplementary Figure 3: protein-protein interaction network of proteins exclusively expressed in no-HS-conditioned melanoma-derived lysates samples. Supplementary Table 1: currently known functions of selected gene-proteins downregulated by HS. Supplementary Table 2: currently known functions of gene-proteins exclusively expressed by non-HS samples. Supplementary list of references. [file 3982942.f1.pdf]

Supplementary figures

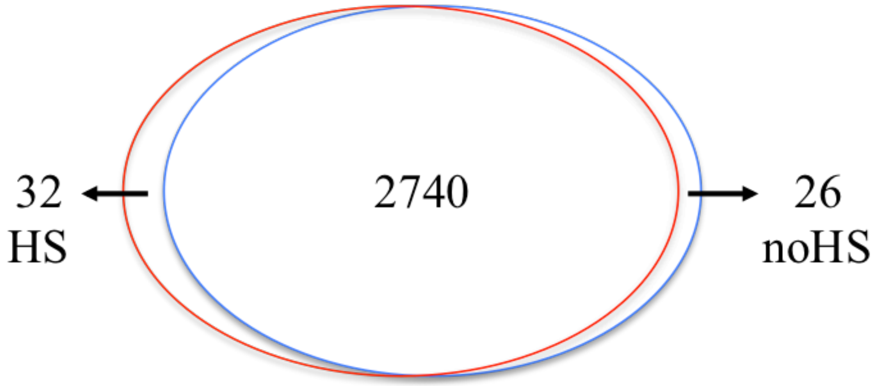

Supplementary Figure 1. Venn-diagram illustrating the number of proteins that overlap between HS and no-HS samples and proteins exclusively identified in HS stimulated samples (HS) and unstimulated (noHS) samples.

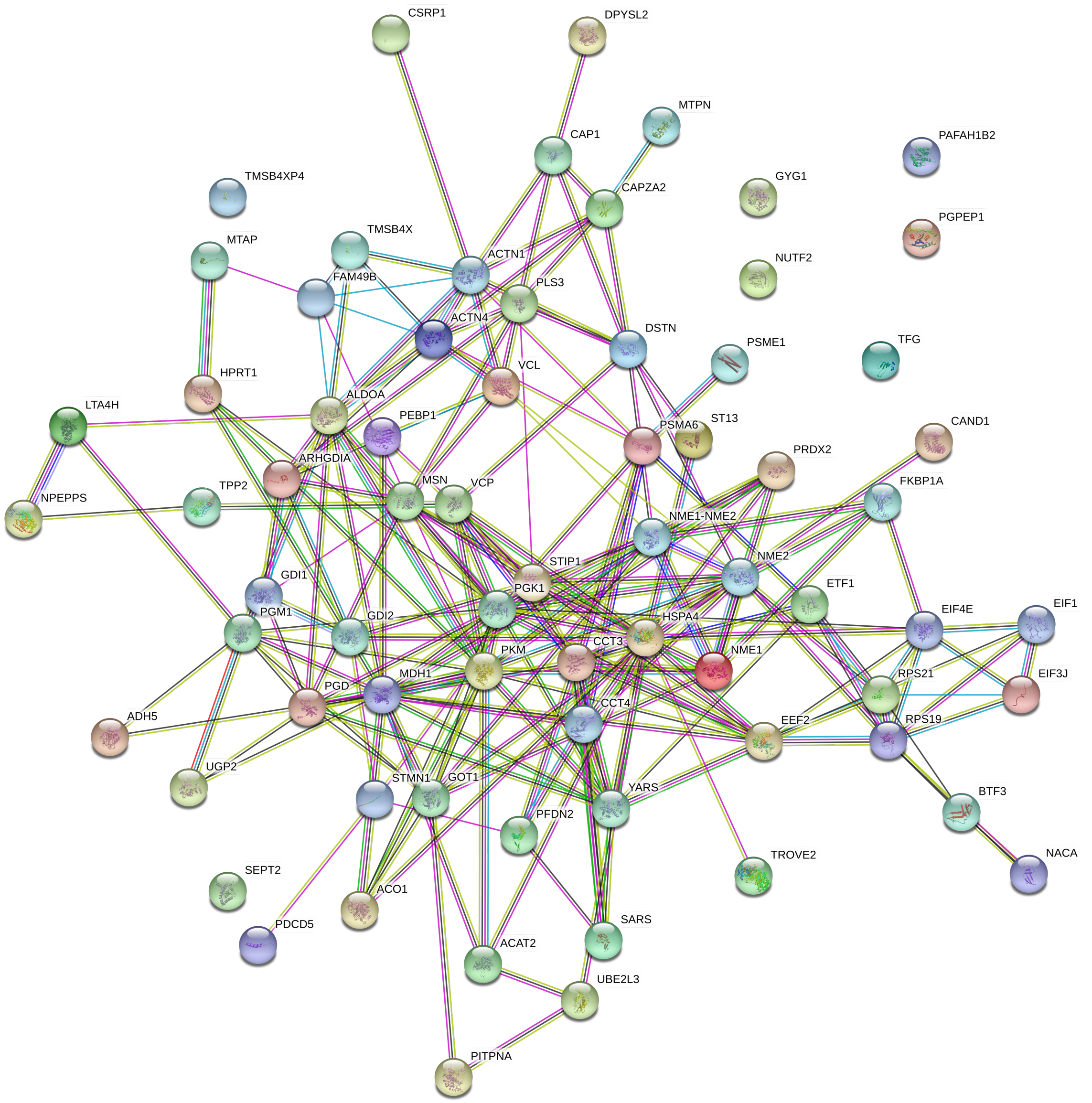

Supplementary Figure 2. Protein-protein interaction network from down-regulated proteins by HS in the lysate TRIMEL. STRING analysis did not find the proteins ST13P4 and ST13P5 (Gene IDs: 145165; 144106), which are not depicted. Line color indicate the type of interaction evidence. Known interactions: cyan, from curated databases; pink, experimentally determined. Predicted interactions: green, gene neighborhood; red, gene fusions; blue; gene co-ocurrence. Others: yellow, textminig; black, co-expression; purple, protein homology. Interaction confidence score, 0.4 (medium).

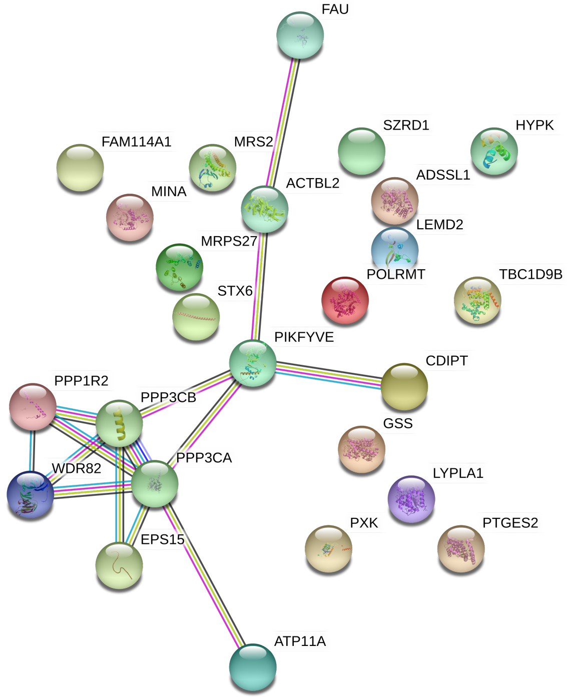

Supplementary Figure 3. Protein-protein interaction network of proteins exclusively expressed in no-HS conditioned melanoma-derived lysates samples. STRING analysis did not find the protein PPP1R2P3 (Gene ID: 153743), which is not depicted. Line color indicate the type of interaction evidence. Known interactions: cyan, from curated databases; pink, experimentally determined. Predicted interactions: green, gene neighborhood; red, gene fusions; blue; gene co-ocurrence. Others: yellow, textminig; black, co-expression; purple, protein homology. Interaction confidence score, 0.4 (medium).

Supplementary tables

Supplementary Table 1. Currently known functions of selected gene-proteins down-regulated by HS. The protein list was filtered by p<0.01 or abs(log2(FC))>1.

Gen	ID (NCBI)	Full name (NCBI)	Function (Gene Ontology)	Reference
ACAT2	39	*Acetyl-CoA acetyltransferase 2*	·Acetyl-CoA C-acetyltransferase activity<br>·Protein binding	[1,2]
ACO1	48	*Aconitase 1*	·Aconitate hydratase activity<br>·3 iron, 4 sulfur cluster; 4 iron, 4 sulfur cluster; RNA; iron-responsive element and protein binding	[3-8]
ACTN1	87	*Actinin alpha 1*	·Ligand-dependent nuclear receptor transcription coactivator and protein homodimerization activity<br>·Actin filament; double-stranded RNA; integrin; ion channel; protein and vinculin binding	[9-14]
ACTN4	81	*Actinin alpha 4*	·Ligand-dependent nuclear receptor transcription coactivator and protein homodimerization activity<br>·RNA; RNA polymerase II regulatory region sequence-specific DNA; actin; actin filament; chromatin DNA; integrin; ion channel; nuclear hormone receptor; nucleoside; protein and retinoic acid receptor binding	[9,12,13,15-19]
ADH5	128	*Alcohol dehydrogenase 5 (class III), chi polypeptide*	·S-(hydroxymethyl)glutathione dehydrogenase; electron transfer and formaldehyde dehydrogenase activity<br>·Fatty acid and zinc ion binding	[20,21]
ALDOA	226	*Aldolase, fructose-bisphosphate A*	·Fructose-bisphosphate aldolase activity<br>·RNA; actin; cadherin; cytoskeletal protein; fructose; identical protein; protein and tubulin binding	[22-28]
ARHGDIA	396	*Rho GDP dissociation inhibitor alpha*	·Protein binding	[29]
BTF3	689	*Basic transcription factor 3*	·RNA and protein binding	[2,15,23]
CAND1	55832	*Cullin associated and neddylation dissociated 1*	·Protein binding	[30]
CAP1	10487	*Cyclase associated actin cytoskeleton regulatory protein 1*		
CAPZA2	830	*Capping actin protein of muscle Z-line alpha subunit 2*		
CCT3	7203	*Chaperonin containing TCP1 subunit 3*	·RNA; protein and protein binding involved in protein folding	[2,23,31]
CCT4	10575	*Chaperonin containing TCP1 subunit 4*	·RNA and protein binding	[15,32]
CSRP1	1465	*Cysteine and glycine rich protein 1*	·RNA and zinc ion binding	[15,33]
DPYSL2	1808	*Dihydropyrimidinase like 2*	·Dihydropyrimidinase activity<br>·Identical protein and protein binding	[2,34]
DSTN	11034	*Destrin, actin depolymerizing factor*	·Actin filament and protein binding	[2,35]
EEF2	1938	*Eukaryotic translation elongation factor 2*	·RNA; cadherin; protein and protein kinase binding	[15,23,25,36-38]
EIF1	10209	*Eukaryotic translation initiation factor 1*	·Translation initiation factor activity<br>·RNA and translation factor activity, RNA binding	[23,39,40]
EIF3J	8669	*Eukaryotic translation initiation factor 3 subunit J*	·Contributes to translation initiation factor activity<br>·Protein binding	[41,42]
EIF4E	1977	*Eukaryotic translation initiation factor 4E*	·Translation initiation factor activity<br>·RNA 7-methylguanosine cap; RNA; RNA cap; enzyme; eukaryotic initiation factor 4G; protein and repressing transcription factor binding	[23,43-47]

(continue on next page)

ETF1	2107	*Eukaryotic translation termination factor 1*	·Translation release factor and translation termination factor activity<br>·RNA; protein; ribosome and sequence-specific mRNA binding	[2,15,48-51]
FAM49B	51571	*Family with sequence similarity 49 member B*	·Protein binding	[2]
FKBP1A	2280	*FK506 binding protein 1A*	·NOT calcium channel inhibitor; peptidyl-prolyl cis-trans isomerase and signal transducer activity<br>·FK506; SMAD; activin; ion channel; macrolide; protein; transforming growth factor beta receptor and type I transforming growth factor beta receptor binding	[2,52-59]
GDI1	2664	*GDP dissociation inhibitor 1*	·GDP-dissociation inhibitor activity<br>·Protein binding	[30,60,61]
GDI2	2665	*GDP dissociation inhibitor 2*	·RNA and protein binding	[23,62]
GOT1	2805	*Glutamic-oxaloacetic transaminase 1*	·L-aspartate:2-oxoglutarate aminotransferase activity	[63,64]
GYG1	2992	*Glycogenin 1*	·Glycogenin glucosyltransferase activity<br>·Protein binding	[2,65]
HPRT1	3251	*Hypoxanthine phosphoribosyltransferase 1*	·Guanine phosphoribosyltransferase; hypoxanthine phosphoribosyltransferase and protein homodimerization activity<br>·Identical protein; magnesium ion and protein binding	[2,61,66-68]
HSPA4	3308	*Heat shock protein family A (Hsp70) member 4*	·ATP and protein binding	[2,69]
LTA4H	4048	*Leukotriene A4 hydrolase*	·Aminopeptidase; epoxide hydrolase; leukotriene-A4 hydrolase; metalloaminopeptidase and peptidase activity<br>·RNA; protein and zinc ion binding	[15,70-74]
MDH1	4190	*Malate dehydrogenase 1*	·Malic enzyme activity<br>·Protein binding	[74,75]
MSN	4478	*Moesin*	·Cell adhesion molecule; double-stranded RNA; enzyme; protein; protein kinase and receptor binding<br>·Structural constituent of cytoskeleton	[11,76-82]
MTAP	4507	*Methylthioadenosine phosphorylase*	·Phosphorylase activity<br>·Protein binding	[2,83]
MTPN	136319	*Myotrophin*		
NACA	4666	*Nascent polypeptide-associated complex alpha subunit*		
NME1/<br>NME1-NME2/<br>NME2	4830/<br>654364/<br>4831	*NME/NM23 nucleoside diphosphate kinase 1/<br>NME1-NME2 readthrough/<br>NME/NM23 nucleoside diphosphate kinase 2*	·Deoxyribonuclease and nucleoside diphosphate kinase activity/-/DNA binding transcription factor and nucleoside diphosphate kinase activity<br>·ATP; GTP; RNA; identical protein; magnesium ion; protein and ribosomal small subunit binding/-/Protein binding	[2,15,61,84-86]/-/[85,87,88]
NPEPPS	9520	*Aminopeptidase puromycin sensitive*	·Aminopeptidase activity	[89]
NUTF2	10204	*Nuclear transport factor 2*	·Nucleocytoplasmic transporter activity<br>·Ran GTPase; identical protein and protein binding	[2,90-92]
PAFAH1B2	5049	*Platelet activating factor acetylhydrolase 1b catalytic subunit 2*	·Protein binding	[93]
PDCD5	9141	*Programmed cell death 5*	·Acetyltransferase activator activity<br>·Beta-tubulin; heparin and protein binding	[94-96]
PEBP1	5037	*Phosphatidylethanolamine binding protein 1*	·RNA; enzyme; phosphatidylethanolamine; protein and protein kinase binding	[15,95,97]
PFDN2	5202	*Prefoldin subunit 2*	·Protein; protein binding involved in protein folding and unfolded protein binding	[98-100]
PGD	5226	*Phosphogluconate dehydrogenase*	·Phosphogluconate dehydrogenase (decarboxylating) activity	[101]

(continue on next page)

PGM1	5236	*Phosphoglucomutase 1*	·Phosphoglucomutase activity<br>·Magnesium ion and protein binding	[74,102,103]
PGK1	5230	*Phosphoglycerate kinase 1*	·Phosphoglycerate kinase and protein-disulfide reductase activity<br>·Protein binding	[104-106]
PGPEP1	54858	*Pyroglutamyl-peptidase I*		
PITPNA	5306	*Phosphatidylinositol transfer protein alpha*	·Phosphatidylcholine transporter and phosphatidylinositol transporter activity<br>·Phosphatidylcholine; phosphatidylglycerol and phosphatidylinositol binding	[107]
PKM	5315	*Pyruvate kinase M1/2*	·Pyruvate kinase activity<br>·MHC class II protein complex; RNA; cadherin and protein binding	[15,25,108-110]
PLS3	5358	*Plastin 3*		
PRDX2	7001	*Peroxiredoxin 2*	·Antioxidant and thioredoxin peroxidase activity<br>·Protein binding	[111-113]
PSMA6	5687	*Proteasome subunit alpha 6*	·Endopeptidase activity<br>·NF-kappaB; RNA; protein and purine ribonucleoside triphosphate binding	[2,114,115]
PSME1	5720	*Proteasome activator subunit 1*	·Protein binding	[93]
RPS19	6223	*Ribosomal protein S19*	·Protein homodimerization activity<br>·RNA; fibroblast growth factor; protein and protein kinase binding<br>·Structural constituent of ribosome	[23,116-120]
RPS21	6227	*Ribosomal protein S21*	·RNA and protein N-terminus binding	[15,121]
SARS	6301	*Seryl-tRNA synthetase*	·Protein homodimerization and serine-tRNA ligase activity<br>·RNA; core promoter sequence-specific DNA and protein binding	[122-125]
SEPT2	4735	*Septin 2*	·Cadherin and protein binding	[25,126]
ST13/<br>ST13P4/<br>ST13P5	6767/<br>145165/<br>144106	*ST13, Hsp70 interacting protein/*<br>*ST13, Hsp70 interacting protein pseudogene 4/*<br>*ST13, Hsp70 interacting protein pseudogene 5*	·Protein binding<br>·Protein binding, bridging	[127,128]
STIP1	10963	*Stress induced phosphoprotein 1*	·RNA and protein binding	[15,129]
STMN1	3925	*Stathmin 1*	·Signal transducer activity<br>·Protein and tubulin binding	[2,130,131]
TFG	10342	*TRK-fused gene*	·Signal transducer activity<br>·Identical protein and protein binding	[54,132,133]
TMSB4X/<br>TMSB4XP4	7114/<br>7118	*Thymosin beta 4, X-linked/*<br>*Thymosin beta 4, X-linked pseudogene 4*	·RNA; actin monomer; enzyme and protein binding	[15,134-136]
TPP2	7174	*Tripeptidyl peptidase 2*	·Aminopeptidase and endopeptidase activity<br>·Identical protein and protein binding	[61,89,137,138]
TROVE2	6738	*TROVE domain family member 2*	·RNA binding	[139]
UBE2L3	7332	*Ubiquitin conjugating enzyme E2 L3*	·Transcription coactivator; ubiquitin conjugating enzyme; ubiquitin-protein transferase activator and ubiquitin-protein transferase activity<br>·RNA; enzyme; protein and ubiquitin protein ligase binding	[15,140-145]
UGP2	7360	*UDP-glucose pyrophosphorylase 2*	·UTP:glucose-1-phosphate uridylyltransferase activity<br>·Identical protein and protein binding	[2,146]

(continue on next page)

VCL	7414	*Vinculin*	·Actin; NOT actin; alpha-catenin; beta-catenin; cadherin; dystroglycan; protein and ubiquitin protein ligase binding	[2,25,147-152]
VCP	7415	*Valosin containing protein*	·ATPase and deubiquitinase activator activity<br>·BAT3 complex; RNA; identical protein; polyubiquitin modification-dependent protein; protein; protein domain specific; protein phosphatase; ubiquitin protein ligase; ubiquitin-like protein ligase and ubiquitin-specific protease binding	[23,153-164]
YARS	8565	*Tyrosyl-tRNA synthetase*	·Signal transducer activity<br>·RNA; interleukin-8 receptor and protein binding	[15,165,166]

Abbreviations: ID, Identification number; NCBI, National Center for Biotechnology Information.

Supplementary Table 2. Currently known functions of gene-proteins exclusive expressed by non-HS samples.

Gen	ID (NCBI)	Full name (NCBI)	Function (Gene Ontology)	Reference
ACTBL2	345651	*Actin, beta like 2*	·Protein binding	[29]
ADSSL1	122622	*Adenylosuccinate synthase like 1*	·Adenylosuccinate synthase activity<br>·GTP and phosphate ion binding	[167,168]
ATP11A	23250	*ATPase phospholipid transporting 11A*	·Protein binding	[169]
CDIPT	10423	*CDP-diacylglycerol--inositol 3-phosphatidyltransferase*	·CDP-diacylglycerol-inositol 3-phosphatidyltransferase activity<br>·Protein binding	[2,170]
EPS15	2060	*Epidermal growth factor receptor pathway substrate 15*	·Cadherin; polyubiquitin and protein binding	[25,171,172]
FAM114A1	92689	*Family with sequence similarity 114 member A1*	·Protein binding	[2]
FAU	2197	*FAU, ubiquitin like and ribosomal protein S30 fusion*	·RNA binding	[23]
GSS	2937	*Glutathione synthetase*	·Protein homodimerization activity<br>·ATP; glutathione; identical protein; magnesium ion and protein binding	[2,74,173]
HYPK	25764	*Huntingtin interacting protein K*	·Protein N-terminus and protein binding	[2,174]
LEMD2	221496	*LEM domain containing 2*	·Protein binding	[175]
LYPLA1	10434	*Lysophospholipase I*	·Lipase; lysophospholipase and palmitoyl-(protein) hydrolase activity	[176-178]
MINA	84864	*Ribosomal oxygenase 2*	·Identical protein binding	[2]
MRPS27	23107	*Mitochondrial ribosomal protein S27*	·Protein binding	[179]
MRS2	57380	*MRS2, magnesium transporter*	·Magnesium ion transmembrane transporter activity	[180]
PIKFYVE	200576	*Phosphoinositide kinase, FYVE-type zinc finger containing*	·1-phosphatidylinositol-4-phosphate 5-kinase activity<br>·Protein binding	[2,181]
POLRMT	5442	*RNA polymerase mitochondrial*	·DNA-directed 5'-3' RNA polymerase activity<br>·RNA and protein binding	[15,23,182]
PPP1R2/<br>PPP1R2P3	5504/<br>153743	*Protein phosphatase 1 regulatory inhibitor subunit 2/<br>Protein phosphatase 1 regulatory inhibitor subunit 2 pseudogene 3*	·Protein serine/threonine phosphatase inhibitor activity<br>·Protein binding/Protein binding	[29,183]/<br>[2]
PPP3CA/<br>PPP3CB	5530/<br>5532	*Protein phosphatase 3 catalytic subunit alpha/<br>Protein phosphatase 3 catalytic subunit beta*	·Calmodulin-dependent protein phosphatase; protein dimerization and protein serine/threonine phosphatase activity/Calmodulin-dependent protein phosphatase; protein dimerization and protein serine/threonine phosphatase activity<br>·Calcium ion; calmodulin; cyclosporin A; drug; enzyme and protein binding/ Calcium ion; calmodulin; drug; enzyme; protein and protein phosphatase 2B binding	[2,184-188]/<br>[185,186,189-191]
PTGES2	80142	*Prostaglandin E synthase 2*	·Protein binding	[2]
PXK	54899	*PX domain containing serine/threonine kinase like*	·NOT protein kinase activity<br>·NOT ATP and NOT nucleotide binding	[192]
STX6	10228	*Syntaxin 6*	·Protein and syntaxin binding	[2,193]

(continue on next page)

SZRD1	26099	*SUZ RNA binding domain containing 1*		
TBC1D9B	23061	*TBC1 domain family member 9B*	·Protein binding	[194]
WDR82	80335	*WD repeat domain 82*	·Contributes to histone methyltransferase activity (H3-K4 specific)<br>·Protein binding	[195,196]

Abbreviations: ID, Identification number; NCBI, National Center for Biotechnology Information.

Supplementary references

1. Song XQ, Fukao T, Yamaguchi S, Miyazawa S, Hashimoto T, et al. (1994) Molecular cloning and nucleotide sequence of complementary DNA for human hepatic cytosolic acetoacetyl-coenzyme A thiolase. Biochem Biophys Res Commun 201: 478-485.
2. Rolland T, Tasan M, Charloteaux B, Pevzner SJ, Zhong Q, et al. (2014) A proteome-scale map of the human interactome network. Cell 159: 1212-1226.
3. Philpott CC, Klausner RD, Rouault TA (1994) The bifunctional iron-responsive element binding protein/cytosolic aconitase: the role of active-site residues in ligand binding and regulation. Proc Natl Acad Sci U S A 91: 7321-7325.
4. Haile DJ, Rouault TA, Harford JB, Kennedy MC, Blondin GA, et al. (1992) Cellular regulation of the iron-responsive element binding protein: disassembly of the cubane iron-sulfur cluster results in high-affinity RNA binding. Proc Natl Acad Sci U S A 89: 11735-11739.
5. Dupuy J, Volbeda A, Carpentier P, Darnault C, Moulis JM, et al. (2006) Crystal structure of human iron regulatory protein 1 as cytosolic aconitase. Structure 14: 129-139.
6. Kaptain S, Downey WE, Tang C, Philpott C, Haile D, et al. (1991) A regulated RNA binding protein also possesses aconitase activity. Proc Natl Acad Sci U S A 88: 10109-10113.
7. Hentze MW, Argos P (1991) Homology between IRE-BP, a regulatory RNA-binding protein, aconitase, and isopropylmalate isomerase. Nucleic Acids Res 19: 1739-1740.
8. Blandin G, Marchand S, Charton K, Daniele N, Gicquel E, et al. (2013) A human skeletal muscle interactome centered on proteins involved in muscular dystrophies: LGMD interactome. Skelet Muscle 3: 3.
9. Khurana S, Chakraborty S, Lam M, Liu Y, Su YT, et al. (2012) Familial focal segmental glomerulosclerosis (FSGS)-linked alpha-actinin 4 (ACTN4) protein mutants lose ability to activate transcription by nuclear hormone receptors. J Biol Chem 287: 12027-12035.
10. Edlund M, Lotano MA, Otey CA (2001) Dynamics of alpha-actinin in focal adhesions and stress fibers visualized with alpha-actinin-green fluorescent protein. Cell Motil Cytoskeleton 48: 190-200.
11. Watanabe A, Tatematsu M, Saeki K, Shibata S, Shime H, et al. (2011) Raftlin is involved in the nucleocapture complex to induce poly(I:C)-mediated TLR3 activation. J Biol Chem 286: 10702-10711.
12. Ziane R, Huang H, Moghadaszadeh B, Beggs AH, Levesque G, et al. (2010) Cell membrane expression of cardiac sodium channel Na(v)1.5 is modulated by alpha-actinin-2 interaction. Biochemistry 49: 166-178.
13. Yang X, Coulombe-Huntington J, Kang S, Sheynkman GM, Hao T, et al. (2016) Widespread Expansion of Protein Interaction Capabilities by Alternative Splicing. Cell 164: 805-817.
14. Bois PR, Borgon RA, Vonrhein C, Izard T (2005) Structural dynamics of alpha-actinin-vinculin interactions. Mol Cell Biol 25: 6112-6122.

15. Castello A, Fischer B, Eichelbaum K, Horos R, Beckmann BM, et al. (2012) Insights into RNA biology from an atlas of mammalian mRNA-binding proteins. Cell 149: 1393-1406.
16. Zhao X, Hsu KS, Lim JH, Bruggeman LA, Kao HY (2015) alpha-Actinin 4 potentiates nuclear factor kappa-light-chain-enhancer of activated B-cell (NF-kappaB) activity in podocytes independent of its cytoplasmic actin binding function. J Biol Chem 290: 338-349.
17. Pavalko FM, LaRoche SM (1993) Activation of human neutrophils induces an interaction between the integrin beta 2-subunit (CD18) and the actin binding protein alpha-actinin. J Immunol 151: 3795-3807.
18. Ehrlicher AJ, Krishnan R, Guo M, Bidan CM, Weitz DA, et al. (2015) Alpha-actinin binding kinetics modulate cellular dynamics and force generation. Proc Natl Acad Sci U S A 112: 6619-6624.
19. Goto H, Wakui H, Komatsuda A, Ohtani H, Imai H, et al. (2003) Renal alpha-actinin-4: purification and puromycin aminonucleoside-binding property. Nephron Exp Nephrol 93: e27-35.
20. Engeland K, Hoog JO, Holmquist B, Estonius M, Jornvall H, et al. (1993) Mutation of Arg-115 of human class III alcohol dehydrogenase: a binding site required for formaldehyde dehydrogenase activity and fatty acid activation. Proc Natl Acad Sci U S A 90: 2491-2494.
21. Wagner FW, Pares X, Holmquist B, Vallee BL (1984) Physical and enzymatic properties of a class III isozyme of human liver alcohol dehydrogenase: chi-ADH. Biochemistry 23: 2193-2199.
22. Esposito G, Vitagliano L, Costanzo P, Borrelli L, Barone R, et al. (2004) Human aldolase A natural mutants: relationship between flexibility of the C-terminal region and enzyme function. Biochem J 380: 51-56.
23. Baltz AG, Munschauer M, Schwanhausser B, Vasile A, Murakawa Y, et al. (2012) The mRNA-bound proteome and its global occupancy profile on protein-coding transcripts. Mol Cell 46: 674-690.
24. Clarke FM, Morton DJ (1976) Aldolase binding to actin-containing filaments. Formation of paracrystals. Biochem J 159: 797-798.
25. Guo Z, Neilson LJ, Zhong H, Murray PS, Zanivan S, et al. (2014) E-cadherin interactome complexity and robustness resolved by quantitative proteomics. Sci Signal 7: rs7.
26. Kusakabe T, Motoki K, Hori K (1997) Mode of interactions of human aldolase isozymes with cytoskeletons. Arch Biochem Biophys 344: 184-193.
27. Petit FM, Serres C, Bourgeon F, Pineau C, Auer J (2013) Identification of sperm head proteins involved in zona pellucida binding. Hum Reprod 28: 852-865.
28. St-Jean M, Izard T, Sygusch J (2007) A hydrophobic pocket in the active site of glycolytic aldolase mediates interactions with Wiskott-Aldrich syndrome protein. J Biol Chem 282: 14309-14315.
29. Boldt K, van Reeuwijk J, Lu Q, Koutroumpas K, Nguyen TM, et al. (2016) An organelle-specific protein landscape identifies novel diseases and molecular mechanisms. Nat Commun 7: 11491.

30. Hein MY, Hubner NC, Poser I, Cox J, Nagaraj N, et al. (2015) A human interactome in three quantitative dimensions organized by stoichiometries and abundances. Cell 163: 712-723.
31. Freund A, Zhong FL, Venteicher AS, Meng Z, Veenstra TD, et al. (2014) Proteostatic control of telomerase function through TRiC-mediated folding of TCAB1. Cell 159: 1389-1403.
32. Bakthavatsalam D, Soung RH, Tweardy DJ, Chiu W, Dixon RA, et al. (2014) Chaperonin-containing TCP-1 complex directly binds to the cytoplasmic domain of the LOX-1 receptor. FEBS Lett 588: 2133-2140.
33. Wang X, Lee G, Liebhaber SA, Cooke NE (1992) Human cysteine-rich protein. A member of the LIM/double-finger family displaying coordinate serum induction with c-myc. J Biol Chem 267: 9176-9184.
34. Hamajima N, Matsuda K, Sakata S, Tamaki N, Sasaki M, et al. (1996) A novel gene family defined by human dihydropyrimidinase and three related proteins with differential tissue distribution. Gene 180: 157-163.
35. Yeoh S, Pope B, Mannherz HG, Weeds A (2002) Determining the differences in actin binding by human ADF and cofilin. J Mol Biol 315: 911-925.
36. Sagnol S, Yang Y, Bessin Y, Allemand F, Hapkova I, et al. (2014) Homodimerization of RBPMS2 through a new RRM-interaction motif is necessary to control smooth muscle plasticity. Nucleic Acids Res 42: 10173-10184.
37. Davydova E, Ho AY, Malecki J, Moen A, Enserink JM, et al. (2014) Identification and characterization of a novel evolutionarily conserved lysine-specific methyltransferase targeting eukaryotic translation elongation factor 2 (eEF2). J Biol Chem 289: 30499-30510.
38. Bottermann K, Reinartz M, Barsoum M, Kotter S, Godecke A (2013) Systematic Analysis Reveals Elongation Factor 2 and alpha-Enolase as Novel Interaction Partners of AKT2. PLoS One 8: e66045.
39. Sheikh MS, Fernandez-Salas E, Yu M, Hussain A, Dinman JD, et al. (1999) Cloning and characterization of a human genotoxic and endoplasmic reticulum stress-inducible cDNA that encodes translation initiation factor 1(eIF1(A121/SUI1)). J Biol Chem 274: 16487-16493.
40. Querol-Audi J, Sun C, Vogan JM, Smith MD, Gu Y, et al. (2013) Architecture of human translation initiation factor 3. Structure 21: 920-928.
41. Zhou M, Sandercock AM, Fraser CS, Ridlova G, Stephens E, et al. (2008) Mass spectrometry reveals modularity and a complete subunit interaction map of the eukaryotic translation factor eIF3. Proc Natl Acad Sci U S A 105: 18139-18144.
42. So J, Pasculescu A, Dai AY, Williton K, James A, et al. (2015) Integrative analysis of kinase networks in TRAIL-induced apoptosis provides a source of potential targets for combination therapy. Sci Signal 8: rs3.
43. Yanagiya A, Suyama E, Adachi H, Svitkin YV, Aza-Blanc P, et al. (2012) Translational homeostasis via the mRNA cap-binding protein, eIF4E. Mol Cell 46: 847-858.
44. Bradrick SS, Gromeier M (2009) Identification of gemin5 as a novel 7-methylguanosine cap-binding protein. PLoS One 4: e7030.

45. Rychlik W, Domier LL, Gardner PR, Hellmann GM, Rhoads RE (1987) Amino acid sequence of the mRNA cap-binding protein from human tissues. Proc Natl Acad Sci U S A 84: 945-949.
46. Gosselin P, Martineau Y, Morales J, Czjzek M, Glippa V, et al. (2013) Tracking a refined eIF4E-binding motif reveals Angel1 as a new partner of eIF4E. Nucleic Acids Res 41: 7783-7792.
47. Yu C, Ji SY, Sha QQ, Dang Y, Zhou JJ, et al. (2016) BTG4 is a meiotic cell cycle-coupled maternal-zygotic-transition licensing factor in oocytes. Nat Struct Mol Biol 23: 387-394.
48. Janzen DM, Geballe AP (2004) The effect of eukaryotic release factor depletion on translation termination in human cell lines. Nucleic Acids Res 32: 4491-4502.
49. Guenet L, Toutain B, Guilleret I, Chauvel B, Deaven LL, et al. (1999) Human release factor eRF1: structural organisation of the unique functional gene on chromosome 5 and of the three processed pseudogenes. FEBS Lett 454: 131-136.
50. Inge-Vechtomov S, Zhouravleva G, Philippe M (2003) Eukaryotic release factors (eRFs) history. Biol Cell 95: 195-209.
51. Chavatte L, Frolova L, Laugaa P, Kisselev L, Favre A (2003) Stop codons and UGG promote efficient binding of the polypeptide release factor eRF1 to the ribosomal A site. J Mol Biol 331: 745-758.
52. George CH, Sorathia R, Bertrand BM, Lai FA (2003) In situ modulation of the human cardiac ryanodine receptor (hRyR2) by FKBP12.6. Biochem J 370: 579-589.
53. Schiene-Fischer C, Yu C (2001) Receptor accessory folding helper enzymes: the functional role of peptidyl prolyl cis/trans isomerases. FEBS Lett 495: 1-6.
54. Matsuda A, Suzuki Y, Honda G, Muramatsu S, Matsuzaki O, et al. (2003) Large-scale identification and characterization of human genes that activate NF-kappaB and MAPK signaling pathways. Oncogene 22: 3307-3318.
55. Bauriedel G, Jabs A, Kraemer S, Nickenig G, Skowasch D (2008) Neointimal expression of rapamycin receptor FK506-binding protein FKBP12: postinjury animal and human in-stent restenosis tissue characteristics. J Vasc Res 45: 173-178.
56. Yamaguchi T, Kurisaki A, Yamakawa N, Minakuchi K, Sugino H (2006) FKBP12 functions as an adaptor of the Smad7-Smurf1 complex on activin type I receptor. J Mol Endocrinol 36: 569-579.
57. Liu FL, Liu TY, Kung FL (2014) FKBP12 regulates the localization and processing of amyloid precursor protein in human cell lines. J Biosci 39: 85-95.
58. Petschnigg J, Groisman B, Kotlyar M, Taipale M, Zheng Y, et al. (2014) The mammalian-membrane two-hybrid assay (MaMTH) for probing membrane-protein interactions in human cells. Nat Methods 11: 585-592.
59. Wang T, Donahoe PK, Zervos AS (1994) Specific interaction of type I receptors of the TGF-beta family with the immunophilin FKBP-12. Science 265: 674-676.
60. Sedlacek Z, Konecki DS, Korn B, Klauck SM, Poustka A (1994) Evolutionary conservation and genomic organization of XAP-4, an Xq28 located gene coding for a human rab GDP-dissociation inhibitor (GDI). Mamm Genome 5: 633-639.

61. Sahni N, Yi S, Taipale M, Fuxman Bass JI, Coulombe-Huntington J, et al. (2015) Widespread macromolecular interaction perturbations in human genetic disorders. Cell 161: 647-660.
62. Zhang B, Zhang T, Wang G, Wang G, Chi W, et al. (2015) GSK3beta-Dzip1-Rab8 cascade regulates ciliogenesis after mitosis. PLoS Biol 13: e1002129.
63. Watazu Y, Uji Y, Sugiuchi H, Okabe H, Murao S (1990) New automated measurement of mitochondrial aspartate aminotransferase with use of protease 401. Clin Chem 36: 687-689.
64. Doyle JM, Schinina ME, Bossa F, Doonan S (1990) The amino acid sequence of cytosolic aspartate aminotransferase from human liver. Biochem J 270: 651-657.
65. Moslemi AR, Lindberg C, Nilsson J, Tajsharghi H, Andersson B, et al. (2010) Glycogenin-1 deficiency and inactivated priming of glycogen synthesis. N Engl J Med 362: 1203-1210.
66. Keough DT, Hockova D, Holy A, Naesens LM, Skinner-Adams TS, et al. (2009) Inhibition of hypoxanthine-guanine phosphoribosyltransferase by acyclic nucleoside phosphonates: a new class of antimalarial therapeutics. J Med Chem 52: 4391-4399.
67. Eads JC, Scapin G, Xu Y, Grubmeyer C, Sacchettini JC (1994) The crystal structure of human hypoxanthine-guanine phosphoribosyltransferase with bound GMP. Cell 78: 325-334.
68. Shi W, Li CM, Tyler PC, Furneaux RH, Grubmeyer C, et al. (1999) The 2.0 A structure of human hypoxanthine-guanine phosphoribosyltransferase in complex with a transition-state analog inhibitor. Nat Struct Biol 6: 588-593.
69. Fathallah DM, Cherif D, Dellagi K, Arnaout MA (1993) Molecular cloning of a novel human hsp70 from a B cell line and its assignment to chromosome 5. J Immunol 151: 810-813.
70. Tholander F, Muroya A, Roques BP, Fournie-Zaluski MC, Thunnissen MM, et al. (2008) Structure-based dissection of the active site chemistry of leukotriene A4 hydrolase: implications for M1 aminopeptidases and inhibitor design. Chem Biol 15: 920-929.
71. Rudberg PC, Tholander F, Andberg M, Thunnissen MM, Haeggstrom JZ (2004) Leukotriene A4 hydrolase: identification of a common carboxylate recognition site for the epoxide hydrolase and aminopeptidase substrates. J Biol Chem 279: 27376-27382.
72. Rudberg PC, Tholander F, Thunnissen MM, Haeggstrom JZ (2002) Leukotriene A4 hydrolase/aminopeptidase. Glutamate 271 is a catalytic residue with specific roles in two distinct enzyme mechanisms. J Biol Chem 277: 1398-1404.
73. Medina JF, Wetterholm A, Radmark O, Shapiro R, Haeggstrom JZ, et al. (1991) Leukotriene A4 hydrolase: determination of the three zinc-binding ligands by site-directed mutagenesis and zinc analysis. Proc Natl Acad Sci U S A 88: 7620-7624.
74. Lee OH, Kim H, He Q, Baek HJ, Yang D, et al. (2011) Genome-wide YFP fluorescence complementation screen identifies new regulators for telomere signaling in human cells. Mol Cell Proteomics 10: M110 001628.
75. Tanaka T, Inazawa J, Nakamura Y (1996) Molecular cloning and mapping of a human cDNA for cytosolic malate dehydrogenase (MDH1). Genomics 32: 128-130.

76. Barreiro O, Yanez-Mo M, Serrador JM, Montoya MC, Vicente-Manzanares M, et al. (2002) Dynamic interaction of VCAM-1 and ICAM-1 with moesin and ezrin in a novel endothelial docking structure for adherent leukocytes. J Cell Biol 157: 1233-1245.
77. Jin H, Song YP, Boel G, Kochar J, Pancholi V (2005) Group A streptococcal surface GAPDH, SDH, recognizes uPAR/CD87 as its receptor on the human pharyngeal cell and mediates bacterial adherence to host cells. J Mol Biol 350: 27-41.
78. Steger M, Tonelli F, Ito G, Davies P, Trost M, et al. (2016) Phosphoproteomics reveals that Parkinson's disease kinase LRRK2 regulates a subset of Rab GTPases. Elife 5.
79. Guaitoli G, Raimondi F, Gilsbach BK, Gomez-Llorente Y, Deyaert E, et al. (2016) Structural model of the dimeric Parkinson's protein LRRK2 reveals a compact architecture involving distant interdomain contacts. Proc Natl Acad Sci U S A 113: E4357-4366.
80. Belkina NV, Liu Y, Hao JJ, Karasuyama H, Shaw S (2009) LOK is a major ERM kinase in resting lymphocytes and regulates cytoskeletal rearrangement through ERM phosphorylation. Proc Natl Acad Sci U S A 106: 4707-4712.
81. Zhang M, Bohlson SS, Dy M, Tenner AJ (2005) Modulated interaction of the ERM protein, moesin, with CD93. Immunology 115: 63-73.
82. Lankes WT, Furthmayr H (1991) Moesin: a member of the protein 4.1-talin-ezrin family of proteins. Proc Natl Acad Sci U S A 88: 8297-8301.
83. Della Ragione F, Takabayashi K, Mastropietro S, Mercurio C, Oliva A, et al. (1996) Purification and characterization of recombinant human 5'-methylthioadenosine phosphorylase: definite identification of coding cDNA. Biochem Biophys Res Commun 223: 514-519.
84. Beresford PJ, Zhang D, Oh DY, Fan Z, Greer EL, et al. (2001) Granzyme A activates an endoplasmic reticulum-associated caspase-independent nuclease to induce single-stranded DNA nicks. J Biol Chem 276: 43285-43293.
85. Chen Y, Gallois-Montbrun S, Schneider B, Veron M, Morera S, et al. (2003) Nucleotide binding to nucleoside diphosphate kinases: X-ray structure of human NDPK-A in complex with ADP and comparison to protein kinases. J Mol Biol 332: 915-926.
86. Kim SH, Kim J (2006) Reduction of invasion in human fibrosarcoma cells by ribosomal protein S3 in conjunction with Nm23-H1 and ERK. Biochim Biophys Acta 1763: 823-832.
87. Postel EH, Berberich SJ, Flint SJ, Ferrone CA (1993) Human c-myc transcription factor PuF identified as nm23-H2 nucleoside diphosphate kinase, a candidate suppressor of tumor metastasis. Science 261: 478-480.
88. Boissan M, Montagnac G, Shen Q, Griparic L, Guitton J, et al. (2014) Membrane trafficking. Nucleoside diphosphate kinases fuel dynamin superfamily proteins with GTP for membrane remodeling. Science 344: 1510-1515.
89. Stoltze L, Schirle M, Schwarz G, Schroter C, Thompson MW, et al. (2000) Two new proteases in the MHC class I processing pathway. Nat Immunol 1: 413-418.
90. Steggerda SM, Black BE, Paschal BM (2000) Monoclonal antibodies to NTF2 inhibit nuclear protein import by preventing nuclear translocation of the GTPase Ran. Mol Biol Cell 11: 703-719.

91. Van Impe K, Hubert T, De Corte V, Vanloo B, Boucherie C, et al. (2008) A new role for nuclear transport factor 2 and Ran: nuclear import of CapG. Traffic 9: 695-707.
92. Lu M, Zak J, Chen S, Sanchez-Pulido L, Severson DT, et al. (2014) A code for RanGDP binding in ankyrin repeats defines a nuclear import pathway. Cell 157: 1130-1145.
93. Rual JF, Venkatesan K, Hao T, Hirozane-Kishikawa T, Dricot A, et al. (2005) Towards a proteome-scale map of the human protein-protein interaction network. Nature 437: 1173-1178.
94. Xiao J, Liu C, Li G, Peng S, Hu J, et al. (2013) PDCD5 negatively regulates autoimmunity by upregulating FOXP3(+) regulatory T cells and suppressing Th17 and Th1 responses. J Autoimmun 47: 34-44.
95. Tracy CM, Gray AJ, Cuellar J, Shaw TS, Howlett AC, et al. (2014) Programmed cell death protein 5 interacts with the cytosolic chaperonin containing tailless complex polypeptide 1 (CCT) to regulate beta-tubulin folding. J Biol Chem 289: 4490-4502.
96. Ling X, Liu Y, Fan H, Zhong Y, Li D, et al. (2007) Studies on interactions of programmed cell death 5 (PDCD5) and its related peptides with heparin by capillary zone electrophoresis. Anal Bioanal Chem 387: 909-916.
97. Zhao J, O'Donnell VB, Balzar S, St Croix CM, Trudeau JB, et al. (2011) 15-Lipoxygenase 1 interacts with phosphatidylethanolamine-binding protein to regulate MAPK signaling in human airway epithelial cells. Proc Natl Acad Sci U S A 108: 14246-14251.
98. Taipale M, Tucker G, Peng J, Krykbaeva I, Lin ZY, et al. (2014) A quantitative chaperone interaction network reveals the architecture of cellular protein homeostasis pathways. Cell 158: 434-448.
99. Tsao ML, Chao CH, Yeh CT (2006) Interaction of hepatitis C virus F protein with prefoldin 2 perturbs tubulin cytoskeleton organization. Biochem Biophys Res Commun 348: 271-277.
100. Vainberg IE, Lewis SA, Rommelaere H, Ampe C, Vandekerckhove J, et al. (1998) Prefoldin, a chaperone that delivers unfolded proteins to cytosolic chaperonin. Cell 93: 863-873.
101. Rippa M, Giovannini PP, Barrett MP, Dallocchio F, Hanau S (1998) 6-Phosphogluconate dehydrogenase: the mechanism of action investigated by a comparison of the enzyme from different species. Biochim Biophys Acta 1429: 83-92.
102. Lee Y, Stiers KM, Kain BN, Beamer LJ (2014) Compromised catalysis and potential folding defects in in vitro studies of missense mutants associated with hereditary phosphoglucomutase 1 deficiency. J Biol Chem 289: 32010-32019.
103. Stiers KM, Kain BN, Graham AC, Beamer LJ (2016) Induced Structural Disorder as a Molecular Mechanism for Enzyme Dysfunction in Phosphoglucomutase 1 Deficiency. J Mol Biol 428: 1493-1505.
104. Fujii H, Krietsch WK, Yoshida A (1980) A single amino acid substitution (Asp leads to Asn) in a phosphoglycerate kinase variant (PGK Munchen) associated with enzyme deficiency. J Biol Chem 255: 6421-6423.

105. Lay AJ, Jiang XM, Kisker O, Flynn E, Underwood A, et al. (2000) Phosphoglycerate kinase acts in tumour angiogenesis as a disulphide reductase. Nature 408: 869-873.
106. Wang S, Jiang B, Zhang T, Liu L, Wang Y, et al. (2015) Insulin and mTOR Pathway Regulate HDAC3-Mediated Deacetylation and Activation of PGK1. PLoS Biol 13: e1002243.
107. Garner K, Hunt AN, Koster G, Somerharju P, Groves E, et al. (2012) Phosphatidylinositol transfer protein, cytoplasmic 1 (PITPNC1) binds and transfers phosphatidic acid. J Biol Chem 287: 32263-32276.
108. Vander Heiden MG, Christofk HR, Schuman E, Subtelny AO, Sharfi H, et al. (2010) Identification of small molecule inhibitors of pyruvate kinase M2. Biochem Pharmacol 79: 1118-1124.
109. Buschow SI, van Balkom BW, Aalberts M, Heck AJ, Wauben M, et al. (2010) MHC class II-associated proteins in B-cell exosomes and potential functional implications for exosome biogenesis. Immunol Cell Biol 88: 851-856.
110. Chen TC, Lin KT, Chen CH, Lee SA, Lee PY, et al. (2014) Using an in situ proximity ligation assay to systematically profile endogenous protein-protein interactions in a pathway network. J Proteome Res 13: 5339-5346.
111. Sanchez-Font MF, Sebastia J, Sanfeliu C, Cristofol R, Marfany G, et al. (2003) Peroxiredoxin 2 (PRDX2), an antioxidant enzyme, is under-expressed in Down syndrome fetal brains. Cell Mol Life Sci 60: 1513-1523.
112. Parmigiani RB, Xu WS, Venta-Perez G, Erdjument-Bromage H, Yaneva M, et al. (2008) HDAC6 is a specific deacetylase of peroxiredoxins and is involved in redox regulation. Proc Natl Acad Sci U S A 105: 9633-9638.
113. Sobotta MC, Liou W, Stocker S, Talwar D, Oehler M, et al. (2015) Peroxiredoxin-2 and STAT3 form a redox relay for H2O2 signaling. Nat Chem Biol 11: 64-70.
114. Bey F, Silva Pereira I, Coux O, Viegas-Pequignot E, Recillas Targa F, et al. (1993) The prosomal RNA-binding protein p27K is a member of the alpha-type human prosomal gene family. Mol Gen Genet 237: 193-205.
115. Bouwmeester T, Bauch A, Ruffner H, Angrand PO, Bergamini G, et al. (2004) A physical and functional map of the human TNF-alpha/NF-kappa B signal transduction pathway. Nat Cell Biol 6: 97-105.
116. Nishimura T, Horino K, Nishiura H, Shibuya Y, Hiraoka T, et al. (2001) Apoptotic cells of an epithelial cell line, AsPC-1, release monocyte chemotactic S19 ribosomal protein dimer. J Biochem 129: 445-454.
117. Soulet F, Al Saati T, Roga S, Amalric F, Bouche G (2001) Fibroblast growth factor-2 interacts with free ribosomal protein S19. Biochem Biophys Res Commun 289: 591-596.
118. Jefferson M, Donaszi-Ivanov A, Pollen S, Dalmay T, Saalbach G, et al. (2014) Host factors that interact with the pestivirus N-terminal protease, Npro, are components of the ribonucleoprotein complex. J Virol 88: 10340-10353.
119. Chiocchetti A, Gibello L, Carando A, Aspesi A, Secco P, et al. (2005) Interactions between RPS19, mutated in Diamond-Blackfan anemia, and the PIM-1 oncoprotein. Haematologica 90: 1453-1462.

120. Yu Y, Ji H, Doudna JA, Leary JA (2005) Mass spectrometric analysis of the human 40S ribosomal subunit: native and HCV IRES-bound complexes. Protein Sci 14: 1438-1446.
121. Sato M, Saeki Y, Tanaka K, Kaneda Y (1999) Ribosome-associated protein LBP/p40 binds to S21 protein of 40S ribosome: analysis using a yeast two-hybrid system. Biochem Biophys Res Commun 256: 385-390.
122. Wang C, Guo Y, Tian Q, Jia Q, Gao Y, et al. (2015) SerRS-tRNASec complex structures reveal mechanism of the first step in selenocysteine biosynthesis. Nucleic Acids Res 43: 10534-10545.
123. Xu X, Shi Y, Yang XL (2013) Crystal structure of human Seryl-tRNA synthetase and Ser-SA complex reveals a molecular lever specific to higher eukaryotes. Structure 21: 2078-2086.
124. Vincent C, Tarbouriech N, Hartlein M (1997) Genomic organization, cDNA sequence, bacterial expression, and purification of human seryl-tRNA synthase. Eur J Biochem 250: 77-84.
125. Shi Y, Xu X, Zhang Q, Fu G, Mo Z, et al. (2014) tRNA synthetase counteracts c-Myc to develop functional vasculature. Elife 3: e02349.
126. Lu H, Galeano MCR, Ott E, Kaeslin G, Kausalya PJ, et al. (2017) Mutations in DZIP1L, which encodes a ciliary-transition-zone protein, cause autosomal recessive polycystic kidney disease. Nat Genet 49: 1025-1034.
127. Soler-Lopez M, Zanzoni A, Lluis R, Stelzl U, Aloy P (2011) Interactome mapping suggests new mechanistic details underlying Alzheimer's disease. Genome Res 21: 364-376.
128. Prapapanich V, Chen S, Nair SC, Rimerman RA, Smith DF (1996) Molecular cloning of human p48, a transient component of progesterone receptor complexes and an Hsp70-binding protein. Mol Endocrinol 10: 420-431.
129. Yadav L, Tamene F, Goos H, van Drogen A, Katainen R, et al. (2017) Systematic Analysis of Human Protein Phosphatase Interactions and Dynamics. Cell Syst 4: 430-444 e435.
130. Kumar R, Haugen JD (1994) Human and rat osteoblast-like cells express stathmin, a growth-regulatory protein. Biochem Biophys Res Commun 201: 861-865.
131. Honnappa S, Cutting B, Jahnke W, Seelig J, Steinmetz MO (2003) Thermodynamics of the Op18/stathmin-tubulin interaction. J Biol Chem 278: 38926-38934.
132. Yachie N, Petsalaki E, Mellor JC, Weile J, Jacob Y, et al. (2016) Pooled-matrix protein interaction screens using Barcode Fusion Genetics. Mol Syst Biol 12: 863.
133. Kanadome T, Shibata H, Kuwata K, Takahara T, Maki M (2017) The calcium-binding protein ALG-2 promotes endoplasmic reticulum exit site localization and polymerization of Trk-fused gene (TFG) protein. FEBS J 284: 56-76.
134. Cassimeris L, Safer D, Nachmias VT, Zigmond SH (1992) Thymosin beta 4 sequesters the majority of G-actin in resting human polymorphonuclear leukocytes. J Cell Biol 119: 1261-1270.
135. Freeman KW, Bowman BR, Zetter BR (2011) Regenerative protein thymosin beta-4 is a novel regulator of purinergic signaling. FASEB J 25: 907-915.
136. Brieger A, Adryan B, Wolpert F, Passmann S, Zeuzem S, et al. (2010) Cytoskeletal scaffolding proteins interact with Lynch-Syndrome associated mismatch repair protein MLH1. Proteomics 10: 3343-3355.

137. Geier E, Pfeifer G, Wilm M, Lucchiari-Hartz M, Baumeister W, et al. (1999) A giant protease with potential to substitute for some functions of the proteasome. Science 283: 978-981.
138. Schonegge AM, Villa E, Forster F, Hegerl R, Peters J, et al. (2012) The structure of human tripeptidyl peptidase II as determined by a hybrid approach. Structure 20: 593-603.
139. Deutscher SL, Harley JB, Keene JD (1988) Molecular analysis of the 60-kDa human Ro ribonucleoprotein. Proc Natl Acad Sci U S A 85: 9479-9483.
140. Verma S, Ismail A, Gao X, Fu G, Li X, et al. (2004) The ubiquitin-conjugating enzyme UBCH7 acts as a coactivator for steroid hormone receptors. Mol Cell Biol 24: 8716-8726.
141. David Y, Ziv T, Admon A, Navon A (2010) The E2 ubiquitin-conjugating enzymes direct polyubiquitination to preferred lysines. J Biol Chem 285: 8595-8604.
142. Staropoli JF, McDermott C, Martinat C, Schulman B, Demireva E, et al. (2003) Parkin is a component of an SCF-like ubiquitin ligase complex and protects postmitotic neurons from kainate excitotoxicity. Neuron 37: 735-749.
143. Wenzel DM, Lissounov A, Brzovic PS, Klevit RE (2011) UBCH7 reactivity profile reveals parkin and HHARI to be RING/HECT hybrids. Nature 474: 105-108.
144. Robinson PA, Leek JP, Thompson J, Carr IM, Bailey A, et al. (1995) A human ubiquitin conjugating enzyme, L-UBC, maps in the Alzheimer's disease locus on chromosome 14q24.3. Mamm Genome 6: 725-731.
145. Scott DC, Rhee DY, Duda DM, Kelsall IR, Olszewski JL, et al. (2016) Two Distinct Types of E3 Ligases Work in Unison to Regulate Substrate Ubiquitylation. Cell 166: 1198-1214 e1124.
146. Peng HL, Chang HY (1993) Cloning of a human liver UDP-glucose pyrophosphorylase cDNA by complementation of the bacterial galU mutation. FEBS Lett 329: 153-158.
147. Cerecedo D, Mondragon R, Cisneros B, Martinez-Perez F, Martinez-Rojas D, et al. (2006) Role of dystrophins and utrophins in platelet adhesion process. Br J Haematol 134: 83-91.
148. Johnson RP, Craig SW (1995) F-actin binding site masked by the intramolecular association of vinculin head and tail domains. Nature 373: 261-264.
149. Watabe-Uchida M, Uchida N, Imamura Y, Nagafuchi A, Fujimoto K, et al. (1998) alpha-Catenin-vinculin interaction functions to organize the apical junctional complex in epithelial cells. J Cell Biol 142: 847-857.
150. Peng X, Cuff LE, Lawton CD, DeMali KA (2010) Vinculin regulates cell-surface E-cadherin expression by binding to beta-catenin. J Cell Sci 123: 567-577.
151. Cerecedo D, Cisneros B, Suarez-Sanchez R, Hernandez-Gonzalez E, Galvan I (2008) beta-Dystroglycan modulates the interplay between actin and microtubules in human-adhered platelets. Br J Haematol 141: 517-528.
152. Davison EJ, Pennington K, Hung CC, Peng J, Rafiq R, et al. (2009) Proteomic analysis of increased Parkin expression and its interactants provides evidence for a role in modulation of mitochondrial function. Proteomics 9: 4284-4297.
153. Jerath NU, Crockett CD, Moore SA, Shy ME, Weihl CC, et al. (2015) Rare Manifestation of a c.290 C>T, p.Gly97Glu VCP Mutation. Case Rep Genet 2015: 239167.

154. Laco MN, Cortes L, Travis SM, Paulson HL, Rego AC (2012) Valosin-containing protein (VCP/p97) is an activator of wild-type ataxin-3. PLoS One 7: e43563.
155. Wang Q, Liu Y, Soetandyo N, Baek K, Hegde R, et al. (2011) A ubiquitin ligase-associated chaperone holdase maintains polypeptides in soluble states for proteasome degradation. Mol Cell 42: 758-770.
156. Hanzelmann P, Schindelin H (2016) Structural Basis of ATP Hydrolysis and Intersubunit Signaling in the AAA+ ATPase p97. Structure 24: 127-139.
157. Banerjee S, Bartesaghi A, Merk A, Rao P, Bulfer SL, et al. (2016) 2.3 A resolution cryo-EM structure of human p97 and mechanism of allosteric inhibition. Science 351: 871-875.
158. Schuller JM, Beck F, Lossl P, Heck AJ, Forster F (2016) Nucleotide-dependent conformational changes of the AAA+ ATPase p97 revisited. FEBS Lett 590: 595-604.
159. Dai RM, Li CC (2001) Valosin-containing protein is a multi-ubiquitin chain-targeting factor required in ubiquitin-proteasome degradation. Nat Cell Biol 3: 740-744.
160. Papadopoulos C, Kirchner P, Bug M, Grum D, Koerver L, et al. (2017) VCP/p97 cooperates with YOD1, UBXD1 and PLAA to drive clearance of ruptured lysosomes by autophagy. EMBO J 36: 135-150.
161. McNeill H, Knebel A, Arthur JS, Cuenda A, Cohen P (2004) A novel UBA and UBX domain protein that binds polyubiquitin and VCP and is a substrate for SAPKs. Biochem J 384: 391-400.
162. Zhang SH, Liu J, Kobayashi R, Tonks NK (1999) Identification of the cell cycle regulator VCP (p97/CDC48) as a substrate of the band 4.1-related protein-tyrosine phosphatase PTPH1. J Biol Chem 274: 17806-17812.
163. Blount JR, Burr AA, Denuc A, Marfany G, Todi SV (2012) Ubiquitin-specific protease 25 functions in Endoplasmic Reticulum-associated degradation. PLoS One 7: e36542.
164. Ye Y, Shibata Y, Kikkert M, van Voorden S, Wiertz E, et al. (2005) Recruitment of the p97 ATPase and ubiquitin ligases to the site of retrotranslocation at the endoplasmic reticulum membrane. Proc Natl Acad Sci U S A 102: 14132-14138.
165. Wakasugi K, Schimmel P (1999) Two distinct cytokines released from a human aminoacyl-tRNA synthetase. Science 284: 147-151.
166. Sajish M, Schimmel P (2015) A human tRNA synthetase is a potent PARP1-activating effector target for resveratrol. Nature 519: 370-373.
167. Park HJ, Hong YB, Choi YC, Lee J, Kim EJ, et al. (2016) ADSSL1 mutation relevant to autosomal recessive adolescent onset distal myopathy. Ann Neurol 79: 231-243.
168. Sun H, Li N, Wang X, Chen T, Shi L, et al. (2005) Molecular cloning and characterization of a novel muscle adenylosuccinate synthetase, AdSSL1, from human bone marrow stromal cells. Mol Cell Biochem 269: 85-94.
169. Takatsu H, Baba K, Shima T, Umino H, Kato U, et al. (2011) ATP9B, a P4-ATPase (a putative aminophospholipid translocase), localizes to the trans-Golgi network in a CDC50 protein-independent manner. J Biol Chem 286: 38159-38167.
170. Lykidis A, Jackson PD, Rock CO, Jackowski S (1997) The role of CDP-diacylglycerol synthetase and phosphatidylinositol synthase activity levels in the

regulation of cellular phosphatidylinositol content. J Biol Chem 272: 33402-33409.
171. Yuan WC, Lee YR, Lin SY, Chang LY, Tan YP, et al. (2014) K33-Linked Polyubiquitination of Coronin 7 by Cul3-KLHL20 Ubiquitin E3 Ligase Regulates Protein Trafficking. Mol Cell 54: 586-600.
172. Shimada A, Yamaguchi A, Kohda D (2016) Structural basis for the recognition of two consecutive mutually interacting DPF motifs by the SGIP1 mu homology domain. Sci Rep 6: 19565.
173. Polekhina G, Board PG, Gali RR, Rossjohn J, Parker MW (1999) Molecular basis of glutathione synthetase deficiency and a rare gene permutation event. EMBO J 18: 3204-3213.
174. Raychaudhuri S, Sinha M, Mukhopadhyay D, Bhattacharyya NP (2008) HYPK, a Huntingtin interacting protein, reduces aggregates and apoptosis induced by N-terminal Huntingtin with 40 glutamines in Neuro2a cells and exhibits chaperone-like activity. Hum Mol Genet 17: 240-255.
175. Gu M, LaJoie D, Chen OS, von Appen A, Ladinsky MS, et al. (2017) LEM2 recruits CHMP7 for ESCRT-mediated nuclear envelope closure in fission yeast and human cells. Proc Natl Acad Sci U S A 114: E2166-E2175.
176. Koyama K, Ogura Y, Nakai D, Watanabe M, Munemasa T, et al. (2014) Identification of bioactivating enzymes involved in the hydrolysis of laninamivir octanoate, a long-acting neuraminidase inhibitor, in human pulmonary tissue. Drug Metab Dispos 42: 1031-1038.
177. Wang A, Yang HC, Friedman P, Johnson CA, Dennis EA (1999) A specific human lysophospholipase: cDNA cloning, tissue distribution and kinetic characterization. Biochim Biophys Acta 1437: 157-169.
178. Dekker FJ, Rocks O, Vartak N, Menninger S, Hedberg C, et al. (2010) Small-molecule inhibition of APT1 affects Ras localization and signaling. Nat Chem Biol 6: 449-456.
179. Tang T, Zheng B, Chen SH, Murphy AN, Kudlicka K, et al. (2009) hNOA1 interacts with complex I and DAP3 and regulates mitochondrial respiration and apoptosis. J Biol Chem 284: 5414-5424.
180. Piskacek M, Zotova L, Zsurka G, Schweyen RJ (2009) Conditional knockdown of hMRS2 results in loss of mitochondrial Mg(2+) uptake and cell death. J Cell Mol Med 13: 693-700.
181. Carpenter CL (2004) Btk-dependent regulation of phosphoinositide synthesis. Biochem Soc Trans 32: 326-329.
182. Minczuk M, He J, Duch AM, Ettema TJ, Chlebowski A, et al. (2011) TEFM (c17orf42) is necessary for transcription of human mtDNA. Nucleic Acids Res 39: 4284-4299.
183. Helps NR, Street AJ, Elledge SJ, Cohen PT (1994) Cloning of the complete coding region for human protein phosphatase inhibitor 2 using the two hybrid system and expression of inhibitor 2 in E. coli. FEBS Lett 340: 93-98.
184. Li GD, Zhang X, Li R, Wang YD, Wang YL, et al. (2008) CHP2 activates the calcineurin/nuclear factor of activated T cells signaling pathway and enhances the oncogenic potential of HEK293 cells. J Biol Chem 283: 32660-32668.

185. Brogan IJ, Pravica V, Hutchinson IV (2000) Genetic conservation of the immunophilin-binding domains of human calcineurin A1 and A2. Transpl Immunol 8: 139-141.
186. Muramatsu T, Kincaid RL (1993) Molecular cloning of a full-length cDNA encoding the catalytic subunit of human calmodulin-dependent protein phosphatase (calcineurin A alpha). Biochim Biophys Acta 1178: 117-120.
187. Jin L, Harrison SC (2002) Crystal structure of human calcineurin complexed with cyclosporin A and human cyclophilin. Proc Natl Acad Sci U S A 99: 13522-13526.
188. Itakura M, Watanabe I, Sugaya T, Takahashi M (2014) Direct association of the unique C-terminal tail of transmembrane AMPA receptor regulatory protein gamma-8 with calcineurin. FEBS J 281: 1366-1378.
189. Guerini D, Klee CB (1989) Cloning of human calcineurin A: evidence for two isozymes and identification of a polyproline structural domain. Proc Natl Acad Sci U S A 86: 9183-9187.
190. Kissinger CR, Parge HE, Knighton DR, Lewis CT, Pelletier LA, et al. (1995) Crystal structures of human calcineurin and the human FKBP12-FK506-calcineurin complex. Nature 378: 641-644.
191. Hisamitsu T, Nakamura TY, Wakabayashi S (2012) Na(+)/H(+) exchanger 1 directly binds to calcineurin A and activates downstream NFAT signaling, leading to cardiomyocyte hypertrophy. Mol Cell Biol 32: 3265-3280.
192. Zou X, Qiu G, Chen C, Wu M, Hu Y, et al. (2005) Expression pattern and subcellular localization of five splice isoforms of human PXK. Int J Mol Med 16: 701-707.
193. Wang Y, Tai G, Lu L, Johannes L, Hong W, et al. (2005) Trans-Golgi network syntaxin 10 functions distinctly from syntaxins 6 and 16. Mol Membr Biol 22: 313-325.
194. Popovic D, Akutsu M, Novak I, Harper JW, Behrends C, et al. (2012) Rab GTPase-activating proteins in autophagy: regulation of endocytic and autophagy pathways by direct binding to human ATG8 modifiers. Mol Cell Biol 32: 1733-1744.
195. Lee JH, Tate CM, You JS, Skalnik DG (2007) Identification and characterization of the human Set1B histone H3-Lys4 methyltransferase complex. J Biol Chem 282: 13419-13428.
196. Lee JH, You J, Dobrota E, Skalnik DG (2010) Identification and characterization of a novel human PP1 phosphatase complex. J Biol Chem 285: 24466-24476.
